# Ferroptosis Transcriptional Regulation and Prognostic Impact in Medulloblastoma Subtypes Revealed by RNA-Seq

**DOI:** 10.3390/antiox14010096

**Published:** 2025-01-15

**Authors:** Christophe Desterke, Yuanji Fu, Jenny Bonifacio-Mundaca, Claudia Monge, Pascal Pineau, Jorge Mata-Garrido, Raquel Francés

**Affiliations:** 1 INSERM UMRS-1310, Faculté de Médecine du Kremlin Bicêtre, Université Paris-Saclay, F-94270 Le Kremlin-Bicêtre, France; christophe.desterke@inserm.fr; 2 INSERM, CNRS, Institut Necker Enfants Malades, Université Paris Cité, F-75015 Paris, France; yuanji.fu@inserm.fr; 3 National Tumor Bank, Department of Pathology, National Institute of Neoplastic Diseases, Surquillo 15038, Peru; jenny.bonifacio@upch.pe; 4 Unité Organisation Nucléaire et Oncogenèse, Institut Pasteur, Université Paris Cité, INSERM U993, F-75015 Paris, France; claudia.monge@pasteur.fr (C.M.); pascal.pineau@pasteur.fr (P.P.); 5 Energy & Memory, Brain Plasticity Unit, CNRS, ESPCI Paris, PSL Research University, F-75006 Paris, France

**Keywords:** ferroptosis, prognosis, medulloblastoma, RNA-sequencing, response to oxidative stress, methionine–cysteine catabolism, epigenetic

## Abstract

Medulloblastoma (MB) is the most common malignant brain tumor in children, typically arising during infancy and childhood. Despite multimodal therapies achieving a response rate of 70% in children older than 3 years, treatment remains challenging. Ferroptosis, a form of regulated cell death, can be induced in medulloblastoma cells in vitro using erastin or RSL3. Using two independent medulloblastoma RNA-sequencing cohorts (MB-PBTA and MTAB-10767), we investigated the expression of ferroptosis-related molecules through multiple approaches, including Weighted Gene Co-Expression Network Analysis (WGCNA), molecular subtype stratification, protein–protein interaction (PPI) networks, and univariable and multivariable overall survival analyses. A prognostic expression score was computed based on a cross-validated ferroptosis signature. In training and validation cohorts, the regulation of the ferroptosis transcriptional program distinguished the four molecular subtypes of medulloblastoma. WGCNA identified nine gene modules in the MB tumor transcriptome; five correlated with molecular subtypes, implicating pathways related to oxidative stress, hypoxia, and trans-synaptic signaling. One module, associated with disease recurrence, included epigenetic regulators and nucleosome organizers. Univariable survival analyses identified a 45-gene ferroptosis prognostic signature associated with nutrient sensing, cysteine and methionine metabolism, and trans-sulfuration within a one-carbon metabolism. The top ten unfavorable ferroptosis genes included *CCT3*, *SNX5*, *SQOR*, *G3BP1*, *CARS1*, *SLC39A14*, *FAM98A*, *FXR1*, *TFAP2C*, and *ATF4*. Patients with a high ferroptosis score showed a worse prognosis, particularly in the G3 and SHH subtypes. The PPI network highlighted IL6 and CBS as unfavorable hub genes. In a multivariable overall survival model, which included gender, age, and the molecular subtype classification, the ferroptosis expression score was validated as an independent adverse prognostic marker (hazard ratio: 5.8; *p*-value = 1.04 × 10^−9^). This study demonstrates that the regulation of the ferroptosis transcriptional program is linked to medulloblastoma molecular subtypes and patient prognosis. A cross-validated ferroptosis signature was identified in two independent RNA-sequencing cohorts, and the ferroptosis score was confirmed as an independent and adverse prognostic factor in medulloblastoma.

## 1. Introduction

Medulloblastoma is an embryonic tumor arising in the cerebellum and represents the most common malignant brain tumor in children, accounting for approximately 20% of all pediatric central nervous system (CNS) tumors [1]. The tumor typically originates from progenitor cells within the external granular layer of the cerebellum, with genetic and epigenetic abnormalities contributing to tumor initiation and progression. Despite advancements in surgical techniques, radiation therapy, and chemotherapy, the treatment of medulloblastoma remains challenging. The current standard of care involves maximal surgical resection followed by craniospinal irradiation and multi-agent chemotherapy [2,3,4]. While this multimodal therapeutic approach has achieved cure rates of 70–75% in children older than three years, it is associated with significant long-term toxicities, including neurocognitive deficits, endocrinopathies, and hearing loss [5,6]. Furthermore, certain molecular subgroups, particularly Group 3 (G3-MB), have poor prognoses with current therapies [7]. There is a critical need to identify novel therapeutic targets to improve outcomes while minimizing long-term side effects [8,9].

Medulloblastomas are classified into four major molecular subgroups based on their transcriptional and epigenetic profiles: WNT-activated (WNT-MB), SHH-activated (SHH-MB), Group 3 (G3-MB), and Group 4 (G4-MB). These subgroups exhibit distinct genetic drivers, clinical features, and prognostic outcomes [10,11,12]. For example, WNT-MB tumors have the most favorable prognosis, while G3-MB tumors are highly aggressive and associated with MYC amplification [13,14]. This molecular heterogeneity underscores the importance of identifying subgroup-specific therapeutic vulnerabilities to improve patient stratification and treatment outcomes.

One emerging area of interest in cancer research is the role of oxidative stress and antioxidants in the tumor biology. Reactive oxygen species (ROS) are byproducts of cellular metabolism that play a dual role in physiology and pathology. In the CNS, physiological levels of ROS are essential for brain development, including neural stem cell proliferation and differentiation [15]. However, elevated ROS levels can cause oxidative stress, leading to DNA damage, lipid peroxidation, and cell death [16]. Cancer cells often exhibit dysregulated redox homeostasis, balancing ROS production to promote tumor growth while avoiding excessive oxidative stress that would trigger cell death. Antioxidant systems, such as glutathione synthesis and glutathione peroxidase (GPX4) activity, are critical for maintaining this balance and protecting tumor cells from ROS-induced damage [17].

Ferroptosis, a regulated form of cell death driven by iron-dependent lipid peroxidation, is gaining attention as a potential therapeutic target in medulloblastoma. Unlike apoptosis and autophagy, ferroptosis is characterized by the accumulation of lipid peroxides and ROS, leading to cell death [18]. In medulloblastoma, several studies have demonstrated that inducing ferroptosis can reduce the tumor cell viability. For example, treatment with erastin and RSL3, two ferroptosis inducers, has been shown to trigger ferroptosis in medulloblastoma cell lines [19]. Additionally, the oncogene FANCD2, which is overexpressed in SHH-MB, suppresses ferroptosis by reducing the intracellular iron and lipid peroxidation levels. A FANCD2 deficiency leads to increased Fe^2^⁺ accumulation, impaired GPX4 activity, and the activation of ferroptosis, resulting in decreased cell proliferation [20].

Antioxidant pathways play a crucial role in regulating ferroptosis sensitivity. The RNF126-FSP1-CoQ10 pathway, for instance, suppresses ferroptosis by preventing lipid peroxidation. RNF126 is overexpressed in G3-MB tumors and is associated with reduced survival. By ubiquitinating ferroptosis suppressor protein 1 (FSP1), RNF126 regulates ferroptosis sensitivity in these tumors [21]. These findings suggest that antioxidant systems and ferroptosis regulation may serve as therapeutic vulnerabilities in medulloblastoma. Targeting ferroptosis-related pathways could enhance the efficacy of current treatments, particularly in high-risk subgroups such as G3-MB [22,23].

In this study, we investigate the diversity of the ferroptosis transcriptional program in medulloblastoma tumors and its association with molecular subtypes. We explore how processes such as the oxidative stress response, hypoxia, and trans-synaptic signaling contribute to ferroptosis regulation in medulloblastoma. Furthermore, we identify a cross-validated ferroptosis signature that predicts patient prognosis. Patients with high ferroptosis scores exhibit worse outcomes, particularly in the G3 and SHH-activated subtypes. Our findings suggest that ferroptosis-related pathways are critical regulators of medulloblastoma progression and recurrence, and the ferroptosis score may serve as a valuable prognostic tool for patient stratification and therapeutic monitoring.

By elucidating the role of ferroptosis in medulloblastoma, this study highlights potential therapeutic targets that could improve patient outcomes, particularly for those with aggressive subtypes. Future studies should focus on translating these findings into clinical applications to enhance the effectiveness of medulloblastoma treatments.

## 2. Materials and Methods

### 2.1. Public Datasets of RNA-Sequencing

#### 2.1.1. Training Cohort

The training cohort of medulloblastoma tumor RNA-seq data was obtained from the Pediatric Brain Tumor Atlas (PBTA) consortium study [24] via the Pediatric CBioPortal website [25]. After filtering and clinical annotation, the cohort comprised 257 RNA-seq experiments (Table 1).

Most samples were derived from male patients (157 males and 100 females), with an average patient age of 8 years at diagnosis. The mean survival duration was 52 months. Most tumor samples were collected from the posterior fossa, ventricles, or mixed regions. The four molecular subtypes of medulloblastoma (WNT, SHH, Group 3, and Group 4) were well represented within the cohort. Additionally, nearly half of the patients experienced disease progression during follow-up (Table 1).

For preprocessing, RNA-seq quantifications were transformed into pseudocounts, normalized using quantile normalization, and batch effects were adjusted with the ComBat algorithm (Supplementary Figure S1).

#### 2.1.2. Validation Cohort

An independent cohort of medulloblastoma tumor RNA-sequencing data was downloaded from the ArrayExpress database under accession number E-MTAB-10767 [26]. This cohort included 331 RNA-sequencing experiments. Most tumor samples were obtained from male children. The validation cohort also included samples from all four molecular subtypes (Table 2); however, overall survival follow-up data were available only for patients in Groups 3 (G3) and 4 (G4) (Table 2). For preprocessing, RNA-sequencing quantifications were transformed into pseudo-counts, normalized using quantile normalization, and batch effects were corrected with the ComBat algorithm (Supplementary Figure S2).

### 2.2. Normalization of RNA-Sequencing Data

Bioinformatics analyses were conducted using the R software environment (version 4.4.1). Raw RNA-sequencing data were transformed into pseudocounts (log2 + 1) and filtered for positive detection. Quantile normalization was performed using the preprocessCore Bioconductor R package (version 1.66.0) [27]. Ferroptosis-related genes were filtered based on the FerrDbV2 database [28] using the ferroviz R package (version 1.0.0), available at https://github.com/cdesterke/ferroviz (accessed on 7 December 2024) [29]. Following k-means clustering, batch normalization was applied using the sva R package (version 3.52.0) [30].

### 2.3. Weighted Gene Co-Expression Network Analysis

The expression of ferroptosis-related genes was used as input to perform Weighted Gene Co-Expression Network Analysis (WGCNA) on PBTA medulloblastoma tumor samples. These analyses were conducted using the WGCNA R package (version 1.73) [31,32]. Sample clustering was applied to the input matrix to identify and exclude outliers. The soft power threshold was estimated to compute the adjacency matrix and the Topological Overlap Matrix (TOM). Gene modules were detected, and multidimensional scaling was performed to reduce dimensions. Correlation analyses were then conducted to associate gene module distribution with phenotypic traits. Functional enrichment of the identified gene modules was performed using the clusterProfiler R package (version 4.12.6) [33,34], with enrichment analyses conducted on the Gene Ontology Biological Process database [35].

### 2.4. Univariate Survival Analyses

Univariate Cox overall survival analysis was performed on the expression of ferroptosis-related genes in two independent RNA-sequencing cohorts using the loopcolcox R package (version 1.0.0), available at https://github.com/cdesterke/loopcolcox (accessed on 8 December 2024) [36]. A cross-survival signature was generated by intersecting the results from the univariate Cox analyses of the two cohorts (Supplementary Tables S1 and S2). A ferroptosis expression score was calculated as the sum of products between the gene expression values and the corresponding Cox beta coefficients for each ferroptosis gene included in the cross-survival signature, using the following formula:

Score = ∑ (Expression × Cox.beta)

Optimal cutpoint threshold on expression score was determined on overall survival logrank residuals with survminer R package version 0.5.0 [37]. Kaplan–Meier and logrank on overall survival were also performed with survminer R-package.

### 2.5. Protein–Protein Interaction Network

A protein–protein interaction (PPI) network was constructed starting with the ferroptosis cross-signature (45 molecules) using the STRING online proteomic tool (version 12), available at https://string-db.org (accessed on 8 December 2024) [38,39]. After constructing the PPI network, functional enrichment was performed using the Gene Ontology Biological Process (GO::BP) database [35] and the Wikipathways database [40].

### 2.6. Multivariable Survival Modeling

For the PBTA medulloblastoma cohort, comprehensive clinical annotations were available (Table 1). After excluding samples from the WNT subtype (due to all cases having experienced a death event during follow-up), a multivariable overall survival model was constructed using the remaining samples. Ferroptosis expression score categories were incorporated into the Cox multivariable model, along with clinical and epidemiological covariates such as age at diagnosis, gender, and molecular subtypes [41]. The linearity of the Cox residuals for each covariate was assessed by performing global and individual Schoenfeld tests. Model optimization was based on the highest concordance index. After calibrating the model using the rms R package (version 6.8-2, with 500 bootstrap iterations), a nomogram was generated to predict survival at 24 months of follow-up [42].

## 3. Results

### 3.1. Ferroptosis-Related Gene Regulatory Modules Are Associated to the Molecular Phenotype and Prognosis of Medulloblastoma

**Figure 1:**
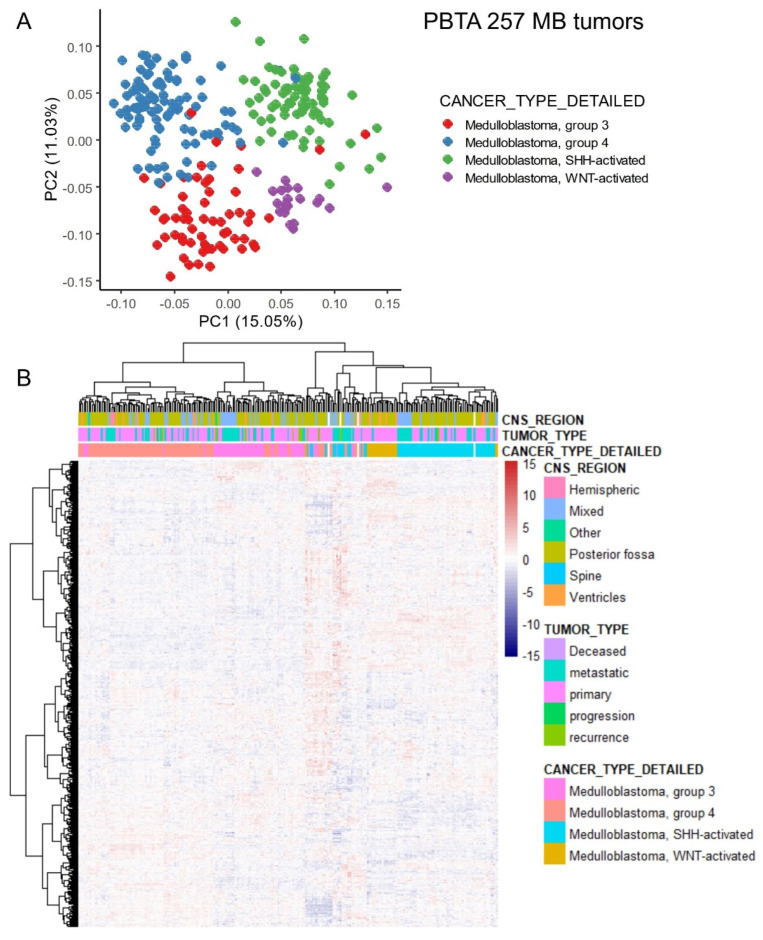
Ferroptosis-related genes stratified the molecular subtypes of medulloblastoma in the PBTA training cohort through unsupervised analyses: (**A**) Principal component analysis (PCA) was performed using the expression profiles of ferroptosis-related genes. (**B**) An expression heatmap and unsupervised hierarchical clustering were generated based on ferroptosis-related gene expression, utilizing Euclidean distances and the Ward.D2 method.

RNA-sequencing data from medulloblastoma samples were filtered from the Pediatric Brain Tumor Atlas (PBTA) cohort [24]. A total of 257 medulloblastoma tumor samples (Table 1) were preprocessed to select ferroptosis-related genes expressed in these tissues (Supplementary Figure S1). Principal component analysis (PCA) based on the expression profiles of 642 ferroptosis-related genes effectively stratified the molecular subtypes of medulloblastoma (G3, G4, WNT, and SHH), as described in previous studies (Figure 1A). This stratification of molecular subtypes was further confirmed by unsupervised clustering using Euclidean distances (Figure 1B). These findings suggest that the transcriptional program of ferroptosis is regulated in accordance with the molecular diversity of medulloblastoma subtypes.

**Figure 2:**
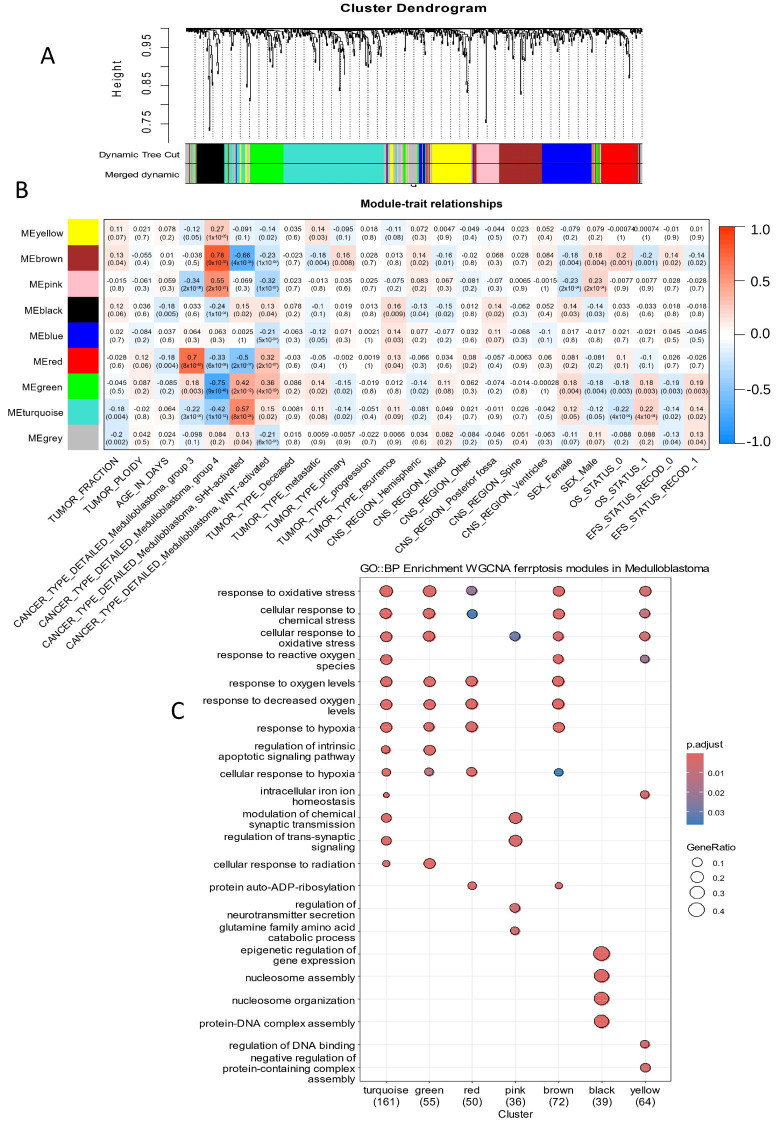
Ferroptosis-related genes identified gene regulatory modules associated with the molecular subtypes and prognosis of medulloblastoma: (**A**) Gene module clustering was performed based on the expression profiles of ferroptosis-related genes. (**B**) Correlation analysis between phenotypic traits and gene regulatory modules, identified from ferroptosis-related gene expression, was conducted. Each intersection cell displays the Pearson correlation coefficient and corresponding *p*-value. (**C**) A dot plot illustrating functional enrichment analysis for each module, performed using the Gene Ontology Biological Process database.

To further investigate the association between the ferroptosis transcriptional program and clinical phenotypes in medulloblastoma, a gene regulatory network (GRN) analysis was conducted using the Weighted Gene Co-Expression Network Analysis (WGCNA) method [31,32]. Sample clustering confirmed the absence of outliers based on ferroptosis-related gene expression in the PBTA medulloblastoma cohort (Supplementary Figure S3A). The optimal network topology, determined by ferroptosis gene expression, identified a soft-threshold power of 16 for initiating the WGCNA analysis (Supplementary Figure S3B). This analysis identified nine independent gene regulatory modules in the ferroptosis transcriptional program of medulloblastoma tumors (Figure 2A and Supplementary Figure S3C). Correlations between these nine gene regulatory modules and clinical phenotypes were assessed (Figure 2B).

The turquoise module was significantly activated in the transcriptomes of SHH subtype tumors (r = 0.57, *p*-value = 8 × 10^−24^, Figure 2B) and in tumors from patients with a positive death status (r = 0.22, *p*-value = 4 × 10^−4^, Figure 2B). The green module was notably repressed in Group 4 tumors (r = −0.75, *p*-value = 0.003, Figure 2B) but activated in tumors from patients with a positive death status (r = 0.18, *p*-value = 4 × 10^−4^, Figure 2B). The red module was prominently activated in the transcriptomes of Group 3 tumors (r = 0.7, *p*-value = 8 × 10^−40^, Figure 2B). The black module was activated in tumors during disease recurrence (r = 0.16, *p*-value = 0.009, Figure 2B). The pink module exhibited significant activation in Group 4 tumors (r = 0.55, *p*-value = 2 × 10^−21^, Figure 2B) and was specifically enriched for genes involved in trans-synaptic signaling (Figure 2C). The brown module was activated in Group 4 tumors (r = 0.78, *p*-value = 9 × 10^−55^, Figure 2B) and repressed in SHH tumors (r = −0.66, *p*-value = 4 × 10^−34^, Figure 2B).

**Figure 3:**
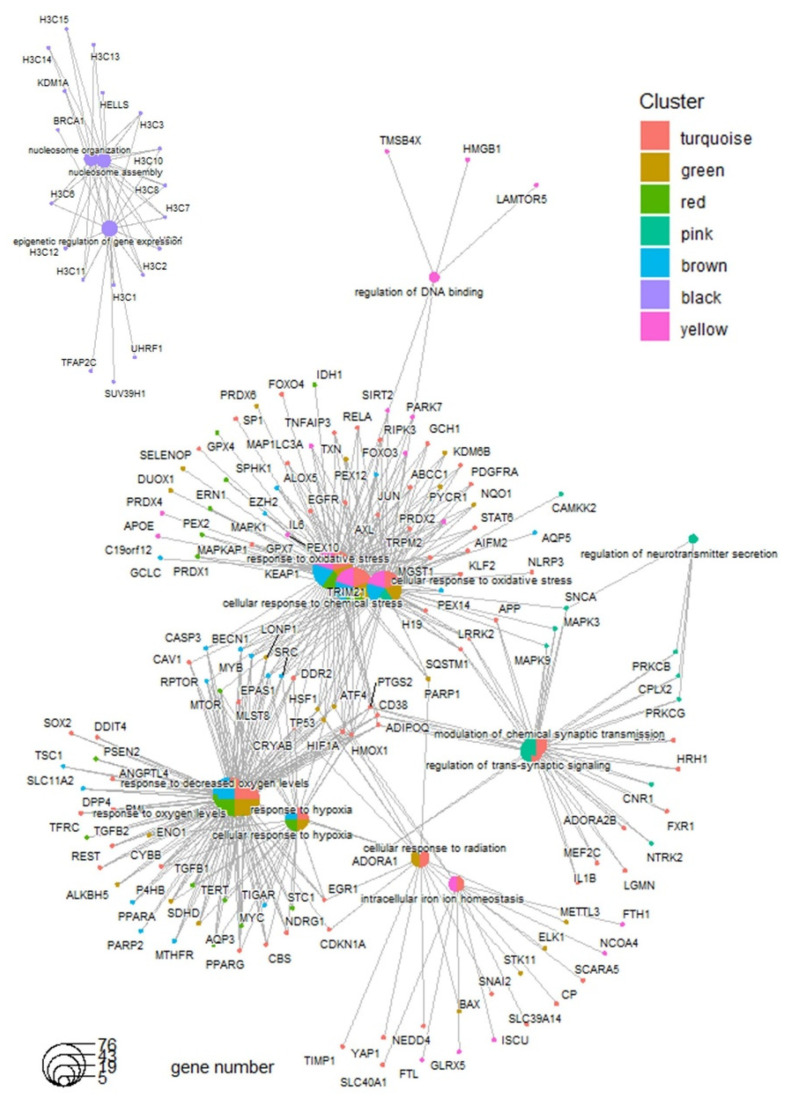
Functional gene network associated with ferroptosis gene regulatory modules detected in tumor of medulloblastoma: enrichment performed by module on gene ontology biological process database.

Functional enrichment analysis of the gene modules using the Gene Ontology Biological Process database revealed that the turquoise, green, red, and brown modules, which are implicated in molecular subtype stratification, shared common functions associated with the response to oxidative stress (Figure 2C and Figure 3).

On the other hand, genes belonging to the black module and associated with the recurrence of the disease (Figure 2B) were found to be enriched in epigenetic regulation and nucleosome organization (Figure 2C and Figure 3). The pink module, upregulated in the Group4 molecular subtype, was enriched in its functionalities, such as the regulation of neurotransmitter secretion and of trans-synaptic signaling (Figure 3). Still, regarding the functional enrichment network (Figure 3), the great majority of ferroptosis genes were shared between the response to oxidative stress (enriched in five gene modules: yellow, brown, red, green, and turquoise, associated with the molecular subtypes) and the response to hypoxia (enriched in four gene modules: brown, red, green, and turquoise, associated with the molecular subtypes). The yellow module (Figure 2), implicated particularly in the response to oxidative stress and not in hypoxia, is specifically upregulated in the Group 4 medulloblastoma subtype. The cellular response to radiation, implicating a smaller enriched network (Figure 3), was found to be enriched in the turquoise and green modules and particularly upregulated in the SHH-medulloblastoma subtype.

### 3.2. Validation of Molecular Sub-Type Stratification by Ferroptosis Transcriptional Program in an Independent Cohort of Medulloblastoma Tumor RNA-Sequencing

**Figure 4:**
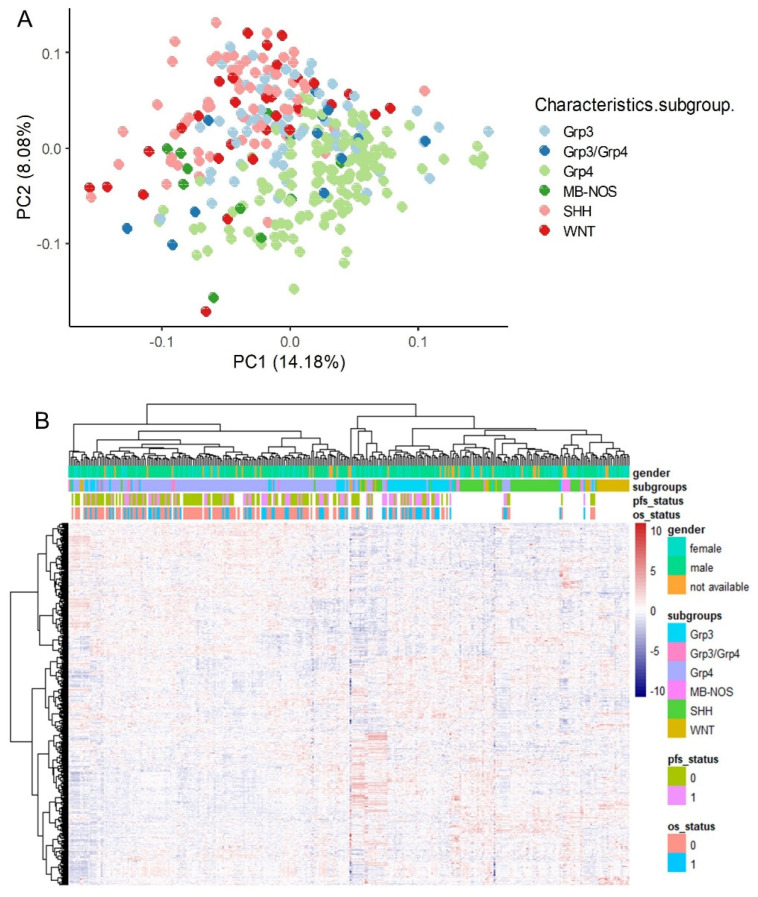
Validation of molecular subtype stratification based on the ferroptosis transcriptional program in an independent cohort of medulloblastoma tumor transcriptomes: (**A**) Principal component analysis (PCA) performed using the expression profiles of ferroptosis-related genes. (**B**) Expression heatmap and unsupervised clustering using Euclidean distances and the Ward.D2 method, based on the ferroptosis transcriptional program.

An independent RNA-sequencing cohort comprising 331 medulloblastoma tumor samples (Table 1) [26] was preprocessed prior to the filtration of ferroptosis-expressed genes (Supplementary Figure S2). Unsupervised principal component analysis (PCA) confirmed the effective stratification of the molecular subtypes based on the expression of the ferroptosis transcriptional program, although a degree of overlap was observed between the SHH and WNT subtypes (Figure 4A). However, the ferroptosis transcriptional program lacked the resolution to further stratify the intermediary groups (G3–G4) described by Williamson et al. [26] within this cohort (Figure 4A). Hierarchical clustering using Euclidean distances proved more effective than PCA in stratifying the molecular subgroups, demonstrating clear distinctions between the SHH and WNT subtypes (Figure 4B).

### 3.3. Cross-Validated Ferroptosis Signature Associated with the Prognosis of Medulloblastoma

**Figure 5:**
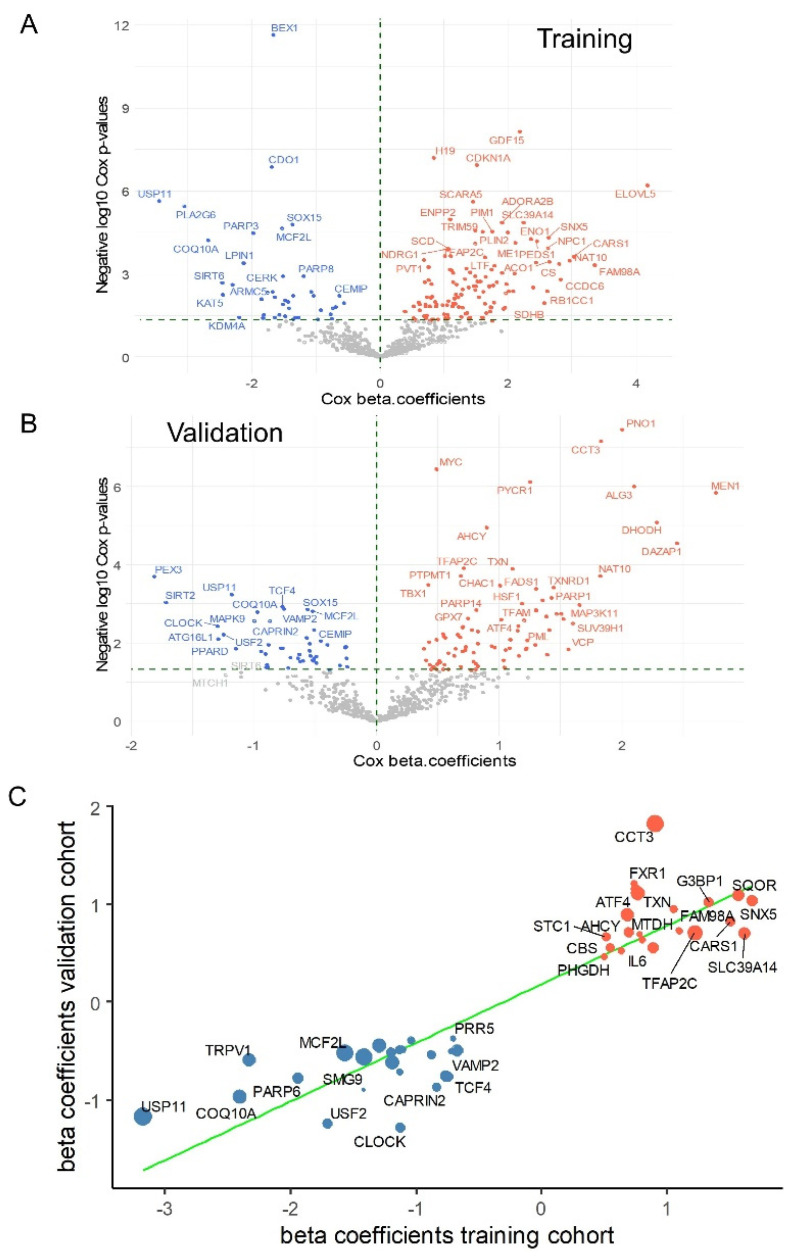
Cross-validated ferroptosis signature associated with the prognosis of medulloblastoma: (**A**) Volcano plot showing beta coefficients versus negative log10 *p*-values from univariate Cox tests performed on the expression of ferroptosis-related genes in the PBTA training cohort. (**B**) Volcano plot showing beta coefficients versus negative log10 *p*-values from univariate Cox tests performed on the expression of ferroptosis-related genes in the validation cohort. (**C**) Scatter plot of Cox beta coefficients from the two independent cohorts for ferroptosis molecules commonly associated with patient prognosis. The size of the dots represents the mean negative log10 Cox *p*-values; tomato-colored dots indicate expression associated with unfavorable prognosis, while blue dots indicate expression associated with favorable prognosis. The green line represents the linear regression fit to the data.

To evaluate the association between ferroptosis gene expression and patient prognosis, iterative Cox proportional hazards analyses were performed with respect to the overall survival in each transcriptomic cohort, focusing on all genes involved in ferroptosis. In the PBTA training cohort, Cox analyses identified 182 ferroptosis-related genes significantly associated with overall survival (Figure 5A, Supplementary Table S1). Similarly, in the validation cohort, Cox analyses identified 133 genes significantly associated with the overall survival (Figure 5B, Supplementary Table S2). A cross-analysis of the univariate survival data from both cohorts revealed a shared signature comprising 45 ferroptosis-related genes whose expression was significantly associated with the prognosis of medulloblastoma patients (Table 3, Figure 5C).

**Figure 6:**
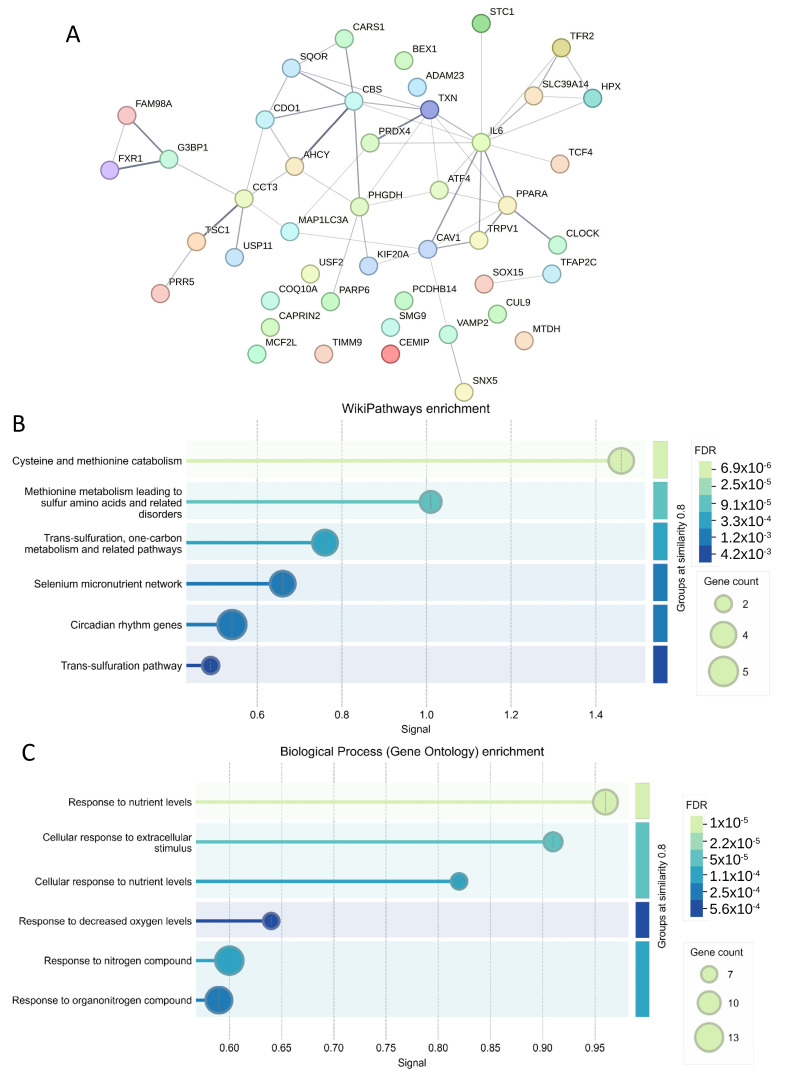
Protein–protein interaction (PPI) network analysis of the ferroptosis cross-validated prognostic signature in medulloblastoma: (**A**) PPI network constructed using edges generated from the STRING proteomics application. (**B**) Functional enrichment analysis of the PPI network using the WikiPathways database. (**C**) Functional enrichment analysis of the PPI network using the Gene Ontology Biological Process database.

Using the prognostic cross-validated ferroptosis signature (45 molecules, Table 3), a protein–protein interaction (PPI) network was constructed using the STRING proteomics online application (Figure 6A). Functional enrichment analysis of the PPI network with the WikiPathways database highlighted key roles in cysteine and methionine catabolism, followed by trans-sulfuration and one-carbon metabolism pathways (Figure 6B). Further functional enrichment analysis using the Gene Ontology Biological Process database revealed significant involvement in cellular responses to nutrient levels, extracellular stimuli, and decreased oxygen levels (Figure 6C).

### 3.4. Computing of a Ferroptosis Expression Score Confirmed Association of Ferroptosis Regulation to Medulloblastoma Prognosis

**Figure 7:**
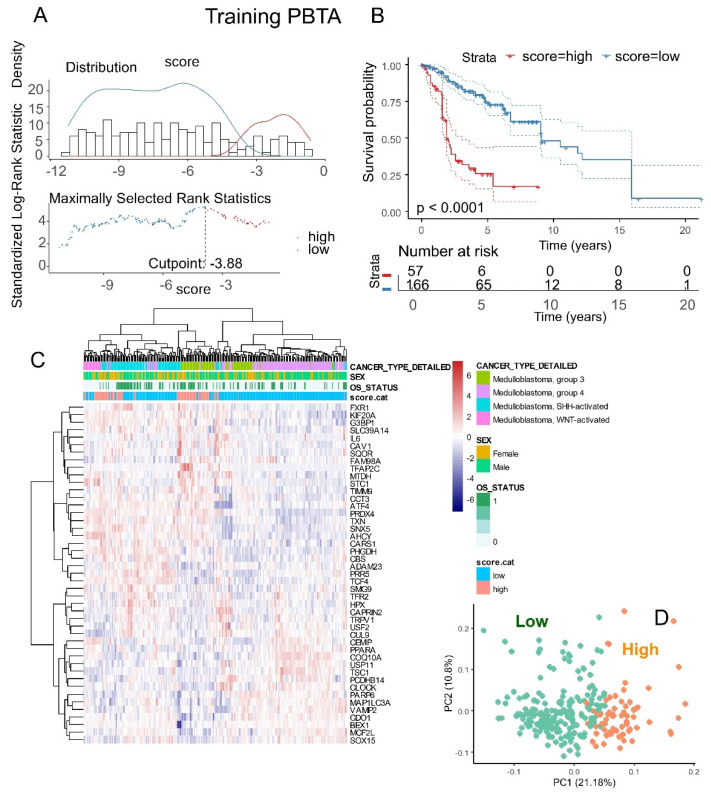
Ferroptosis expression score associated with the overall survival of patients in the PBTA training cohort: (**A**) Optimal cut-point stratification for the ferroptosis expression score based on overall patient survival. (**B**) Kaplan–Meier survival analysis and log-rank test for stratification by ferroptosis score in relation to overall patient survival. (**C**) Expression heatmap and unsupervised clustering (using Euclidean distances) based on the expression profiles of the 45 ferroptosis prognostic molecules. (**D**) Principal component analysis (PCA) based on the expression of the 45 ferroptosis prognostic molecules, with colors indicating ferroptosis expression score categories.

Based on the expression levels and Cox beta coefficients of the 45 ferroptosis molecules from the cross-validated signature (Table 3), a ferroptosis expression score was calculated for two independent cohorts of medulloblastoma tumor transcriptomes. For the PBTA training cohort, an optimal cutoff for the ferroptosis score was determined based on overall patient survival (Figure 7A). Following stratification into two classes, PBTA patients with high ferroptosis scores exhibited significantly worse prognoses compared to those with low scores (log-rank test, *p*-value < 0.0001, Figure 7B). Specifically, patients in the high-ferroptosis group (n = 57) had a median overall survival of 23 months, while those in the low-ferroptosis group (n = 166) had a median overall survival of 109 months.

The expression of the 45 ferroptosis molecules allowed for the identification of a leftward cluster of patients enriched in the high-score category, which included patients with a positive death status, particularly from the SHH and G3 molecular subtypes (Figure 7C). Principal component analysis (PCA) based on the expression of these 45 molecules effectively stratified patients into ferroptosis prognostic categories (Figure 7D).

A higher proportion of female patients was observed in the high-score group compared to the low-score group (*p*-value = 0.016, Table 1). Additionally, the high-score group had significantly higher proportions of progressive metastasis (*p*-value = 0.00016, Table 1). Conversely, the low-score group had a greater proportion of patients from the Group 4 subtype (*p*-value < 1 × 10^−4^, Table 1).

**Figure 8:**
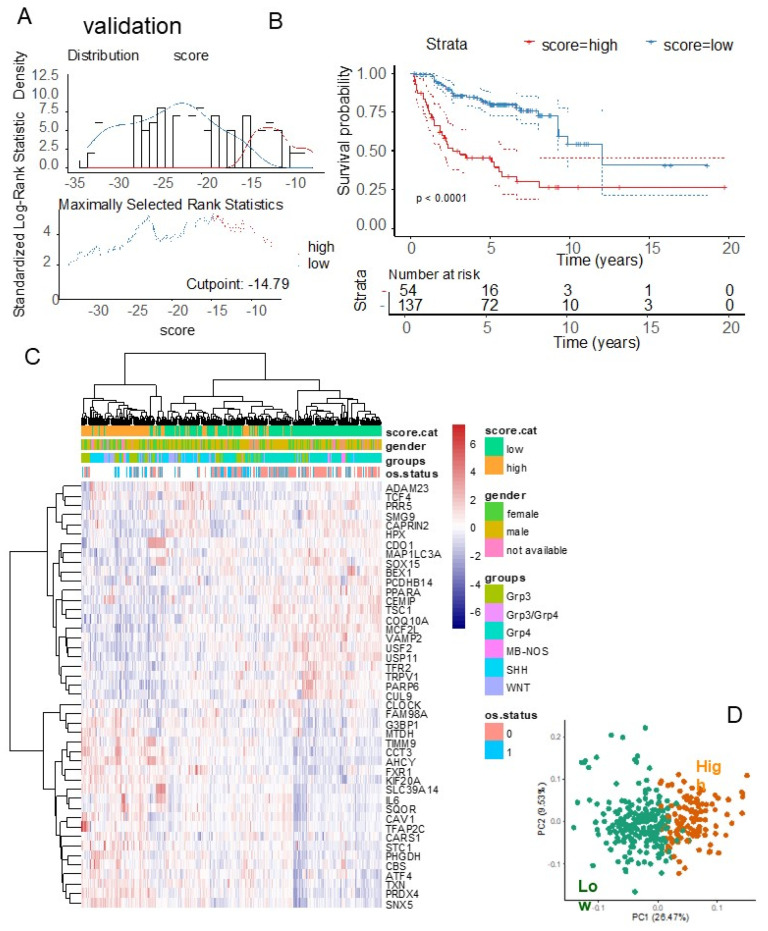
Ferroptosis expression score associated with overall survival of patients in the validation cohort: (**A**) Optimal cut-point stratification for the ferroptosis expression score based on overall patient survival. (**B**) Kaplan–Meier survival analysis and log-rank test for stratification by ferroptosis score in relation to overall survival. (**C**) Expression heatmap and unsupervised clustering (using Euclidean distances) based on the expression profiles of the 45 ferroptosis prognostic molecules. (**D**) Principal component analysis (PCA) based on the expression of the 45 ferroptosis prognostic molecules, with colors indicating ferroptosis expression score categories.

Unsupervised clustering based on the expression of the 45 ferroptosis prognostic molecules further highlighted a leftward cluster comprising patients from the G3 and SHH subtypes and those in the high-ferroptosis prognostic category (Figure 8C, *p*-value < 1 × 10^−4^, Table 2). The ferroptosis prognostic categories were confirmed to be well stratified through PCA in the validation cohort (Figure 8D).

Similarly, in the validation cohort, the ferroptosis expression score was calculated using the expression levels and Cox beta coefficients of the 45 ferroptosis molecules included in the cross-validated signature (Table 3). In this cohort, the overall survival data were available only for patients belonging to the G3 and G4 subtypes [26]. An optimal cut-point for the ferroptosis score based on overall survival was determined for the validation cohort (Figure 8A). A significant difference in prognosis (overall survival) was observed between patients with high ferroptosis scores (n = 54) and those with low ferroptosis scores (n = 137) (log-rank test, *p*-value < 0.0001, Figure 8B). Specifically, high-ferroptosis-score patients in the validation cohort had a median overall survival of 2.68 years, compared to 12.07 years for low-ferroptosis-score patients.

### 3.5. Ferroptosis Expression Score Is an Independent Adverse Prognosis Marker During Medulloblastoma

As the ferroptosis score was strongly associated with patient prognosis in univariate analyses across two independent cohorts, an evaluation of its independence as a prognostic marker was performed through the construction of a multivariable overall survival model. The PBTA medulloblastoma cohort included several annotated clinical parameters (Table 1), and the overall survival information was available for the majority of patients. An optimal cut-point for age at diagnosis was determined, identifying 15 years as the threshold (Supplementary Figure S4A,B). Since no positive death events were observed in patients with the WNT subtype (Supplementary Figure S4C), these patients were excluded from the multivariable analysis.

**Figure 9:**
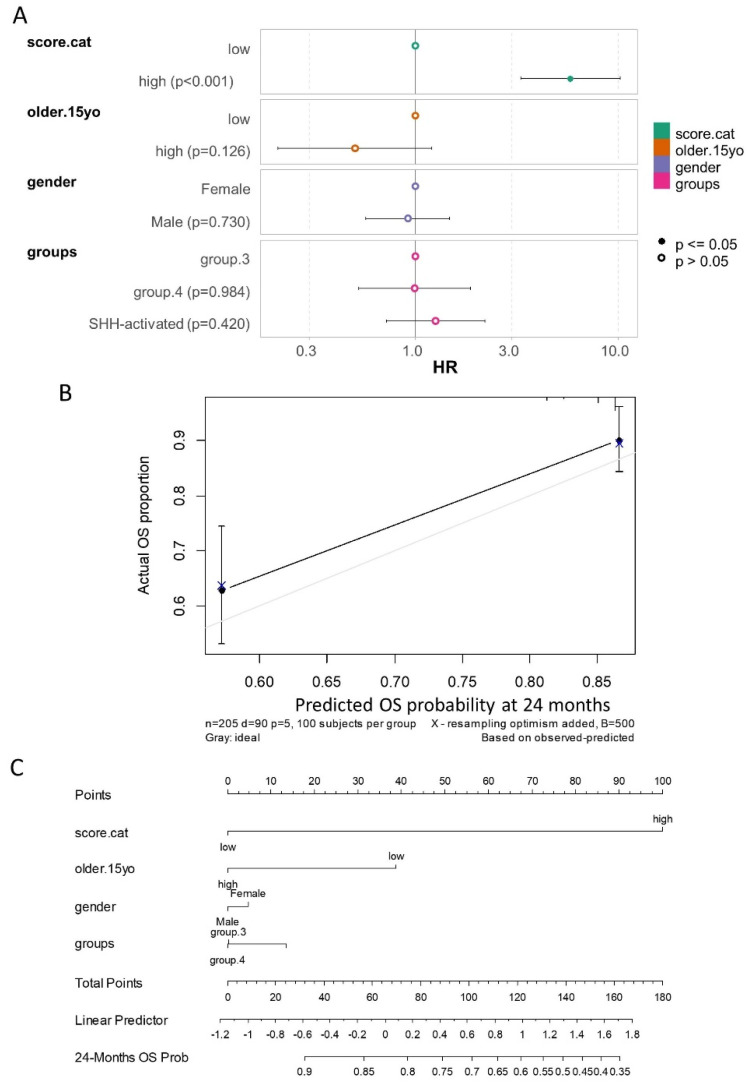
Multivariable overall survival model evaluating the prognosis of medulloblastoma in the PBTA cohort: (**A**) Forest plot of the multivariable overall survival model, including ferroptosis score categories (score.cat), age at diagnosis (older.15yo), gender, and molecular subtypes of patients as covariates (HR: hazard ratios). (**B**) Calibration of the multivariable overall survival model using 500 bootstrap iterations for prediction at 24 months of follow-up. (**C**) Nomogram of the calibrated overall survival model for predicting prognosis at 24 months of follow-up.

The multivariable overall survival model incorporated ferroptosis score categories, age at diagnosis categories, gender, and molecular subtypes. Global and individual Schoenfeld residual tests confirmed the linearity of the residuals for each included covariate (Supplementary Figure S4D). The log-rank test for the multivariable model yielded a highly significant *p*-value (<2 × 10^−16^, Figure 9A). The model demonstrated a concordance index of 0.742 (standard error: 0.026), indicating strong predictive accuracy.

In this multivariable analysis, the ferroptosis expression score was confirmed as an independent adverse prognostic marker (Figure 9A, Table 4), with a hazard ratio of 5.84 (95% confidence interval: 3.31–10.28) for high-score patients compared to low-score patients, and a multivariable *p*-value of 1.04 × 10^−9^.

Following calibration with 500 bootstrap iterations of the multivariable model, prediction stability was achieved at 24 months of follow-up (Figure 9B). The calibrated nomogram of the multivariable overall survival model further emphasized the critical role of the ferroptosis score in predicting patient prognosis at 24 months of follow-up (Figure 9C).

## 4. Discussion

Ferroptosis, an iron-dependent form of non-apoptotic cell death, is characterized by the accumulation of lipid peroxidation products and lethal reactive oxygen species (ROS) generated through iron metabolism [43]. In medulloblastoma, cell radioresistance has been linked to integrin-αvβ3 expression. β3-depleted (β3_KO) medulloblastoma cells exhibit lipid hydroperoxide accumulation after radiotherapy, indicating ferroptosis, a regulated cell death process induced by ROS and inhibited by antioxidants such as cysteine, glutathione (GSH), and glutathione peroxidase 4 (GPx4) [44]. This study found that the regulation of this pathway is associated with the molecular classification of medulloblastoma into four subgroups, involving processes such as cellular responses to oxidative stress and hypoxia and, more specifically, the regulation of trans-synaptic signaling.

Interestingly, ferroptosis plays a critical role in various cancers by influencing tumor microenvironmental interactions, metabolic reprogramming, and immune responses. During medulloblastoma therapy combining the topoisomerase I inhibitor camptothecin (CPT) and the agonistic anti-Fas antibody (CH-11), it was shown that these agents synergize with Fas activation to induce cell death through mechanisms involving ROS and oxidative stress pathways [45]. Consequently, the cellular response to oxidative stress could serve as a potential marker for therapy sensitivity, particularly for treatments that induce cell death. Additionally, oxidative stress plays a dual role in cancer, acting as both a tumor suppressor by inducing cell death and a promoter of tumor progression by activating survival pathways [46,47,48,49]. Hypoxia-inducible factor-1 (HIF-1) activation, driven by mitochondrial ROS formation, shifts the cellular metabolism from oxidative phosphorylation (OXPHOS) to glycolysis by upregulating glycolytic enzymes, thereby promoting tumor progression and metastasis [50].

Part of the ferroptosis transcriptional program identified in this study is implicated in epigenetic regulation and nucleosome organization, both of which are associated with disease recurrence. Mutations in epigenetic regulators affecting H3K27 and H3K4 trimethylation have been reported in G3 and G4 medulloblastoma subtypes [51]. The loss of H3K27 trimethylation contributes to radiotherapy resistance in medulloblastoma and creates a vulnerability that can be targeted with BET inhibition [52]. The link between epigenetic dysregulation and ferroptosis suggests that chromatin remodeling could influence the cellular susceptibility to ferroptosis-inducing therapies, presenting a novel avenue for therapeutic intervention [53,54,55].

A cross-validated ferroptosis signature associated with medulloblastoma prognosis was identified. Patients with high ferroptosis scores exhibited poorer outcomes and were predominantly enriched in the G3 and SHH-activated subtypes. Notably, G3 tumors are associated with poor prognosis, while SHH tumors have an intermediate prognosis [5,56]. Within the protein–protein interaction (PPI) network derived from this signature, IL6 and CBS emerged as central hub genes (Figure 6A) and were also identified as unfavorable markers (Table 3). IL6 has been shown to promote medulloblastoma cell viability, proliferation, and glycolysis [57]. In G3 medulloblastoma, autocrine IL6 signaling activates the STAT3 pathway, contributing to drug resistance; thus, targeting the IL6/STAT3 axis has been proposed as a therapeutic strategy for G3 medulloblastoma [58].

Cystathionine Beta-Synthase (CBS), involved in the trans-sulfuration pathway, facilitates the conversion of homocysteine to cystathionine [59,60]. The PPI network of the cross-validated signature was enriched in processes such as cysteine–methionine catabolism, nutrient response pathways, trans-sulfuration, and one-carbon metabolism. Trans-sulfuration, a key component of one-carbon metabolism, is crucial for converting homocysteine into cystathionine and subsequently into cysteine, which is essential for glutathione synthesis, a potent antioxidant [61]. Nutrient deprivation is a common stressor in the brain tumor microenvironment [62].

Among the unfavorable ferroptosis genes dysregulated in medulloblastoma were *CCT3*, *SNX5*, *SQOR*, *G3BP1*, *CARS1*, *SLC39A14*, *FAM98A*, *FXR1*, *TFAP2C*, and *ATF4* (Table 3). Their roles in ferroptosis have been well-documented. For instance, *CCT3* silencing induces ferroptosis via the NOD1-NF-κB signaling pathway in bladder cancer [63]; SNX5 promotes ferroptosis in Parkinson’s disease, with knockdown experiments reducing intracellular lipid peroxidation and Fe^2^⁺ accumulation [64]; *SQOR* is implicated in selenium-dependent ferroptosis suppression [65]; *G3BP1* mediates the nuclear sequestration of p53, influencing apoptosis and ferroptosis [66]; CARS1 regulates *GPX4* expression to induce ferroptosis [67]; *SLC39A14*, a divalent metal transporter, mediates the manganese, zinc, iron, and cadmium uptake; silencing *SLC39A14* inhibits glioblastoma ferroptosis and progression [68]; FAM98A suppresses ferroptosis, promoting resistance to 5-fluorouracil in colorectal cancer [69]; FXR1 modulates glioma sensitivity to temozolomide by regulating ferroptosis [70]; TFAP2C directly upregulates *GPX4* expression in response to selenium supplementation, reducing brain injury from hemorrhagic stroke [71]; and *ATF4*, a stress-induced transcription factor, orchestrates responses to endoplasmic reticulum stress, amino acid deprivation, and oxidative challenges [72,73].

The potential interplay between ferroptosis-related genes and key signaling pathways like NF-κB, STAT3, and p53 suggests that the ferroptosis transcriptional program could be manipulated to enhance medulloblastoma treatment responses. For instance, targeting the NF-κB pathway has shown promise in sensitizing tumors to ferroptosis-inducing agents [74,75].

Finally, in a multivariable overall survival model incorporating the age, gender, and molecular classification, the ferroptosis score was confirmed as an independent adverse prognostic marker for medulloblastoma. The significance of trans-sulfuration in this context underscores its role in glutathione biosynthesis, providing a mechanistic link to ferroptosis regulation and therapeutic resistance. The emerging evidence suggests that combining ferroptosis-targeting agents with standard therapies may improve patient outcomes by overcoming therapy resistance [76,77].

## 5. Conclusions

This study establishes a strong link between the ferroptosis transcriptional program and medulloblastoma molecular subtypes, identifying a cross-validated ferroptosis signature associated with patient prognosis. High ferroptosis scores were confirmed as independent adverse prognostic markers in two independent cohorts, with IL6 and CBS highlighted as key hub genes driving unfavorable outcomes. Our findings underscore the importance of ferroptosis regulation in medulloblastoma pathogenesis and recurrence, offering valuable insights into subtype-specific vulnerabilities. The ferroptosis score represents a promising tool for patient stratification and therapeutic monitoring, particularly in the context of ferroptosis-targeting interventions.

Future studies should explore the clinical translation of these findings to enhance therapeutic outcomes for medulloblastoma patients. Moreover, understanding the mechanisms driving ferroptosis resistance could pave the way for novel combination therapies targeting epigenetic regulators, metabolic pathways, and immune checkpoints. Such strategies could improve the efficacy of ferroptosis-based interventions in aggressive medulloblastoma subtypes.

## Figures and Tables

**Table 1:** Clinical characteristics from MB patients with processed RNA-sequencing samples in training PBTA cohort: For respective epidemiological parameters, numbers and percentage of patients were described in Total column for qualitative parameter and means/standard deviations for quantitative parameters. Cohort stratification for low and high subtypes was performed on ferroptosis expression score threshold determined during this study. *p*-values test the difference between low- and high-ferroptosis patient subtypes by chi-square test for qualitative parameters and *t*-test for quantitative parameters.

Variable	Level	Low (n = 192)	High (n = 65)	Total (n = 257)	*p*-Value
overall survival time in years	mean (sd)	5 (3.8)	2.3 (1.7)	4.3 (3.6)	<1 × 10^−4^
gender	Male	126 (65.6)	31 (47.7)	157 (61.1)	
	Female	66 (34.4)	34 (52.3)	100 (38.9)	0.0156940
age in years	mean (sd)	8.3 (5.5)	7 (4.9)	8 (5.4)	0.0873753
central nervous system region	Ventricles	12 (6.3)	5 (7.8)	17 (6.7)	
	Mixed	45 (23.8)	21 (32.8)	66 (26.1)	
	Posterior fossa	128 (67.7)	37 (57.8)	165 (65.2)	
	Spine	1 (0.5)	0 (0.0)	1 (0.4)	
	Hemispheric	3 (1.6)	0 (0.0)	3 (1.2)	
	Other	0 (0.0)	1 (1.6)	1 (0.4)	0.2467076
CANCER_TYPE_DETAILED	Medulloblastoma, WNT-activated	10 (5.3)	11 (16.9)	21 (8.3)	
	Medulloblastoma, group 3	34 (18.0)	26 (40.0)	60 (23.6)	
	Medulloblastoma, group 4	96 (50.8)	3 (4.6)	99 (39.0)	
	Medulloblastoma, SHH-activated	49 (25.9)	25 (38.5)	74 (29.1)	<1 × 10^−4^
Event-free survival no_event	0	74 (38.5)	42 (64.6)	116 (45.1)	
	1	118 (61.5)	23 (35.4)	141 (54.9)	0.0004531
Event-free survival deceased_due_to_disease	0	185 (96.4)	63 (96.9)	248 (96.5)	
	1	7 (3.6)	2 (3.1)	9 (3.5)	1.0000000
event-free survival progressive	0	185 (96.4)	65 (100.0)	250 (97.3)	
	1	7 (3.6)	0 (0.0)	7 (2.7)	0.2627078
event-free survival progressive_metastatic	0	176 (91.7)	47 (72.3)	223 (86.8)	
	1	16 (8.3)	18 (27.7)	34 (13.2)	0.0001633
event-free survival recurrence	1	23 (12.0)	14 (21.5)	37 (14.4)	
	0	169 (88.0)	51 (78.5)	220 (85.6)	0.0904289
event-free survival recurrence_metastatic	0	176 (91.7)	58 (89.2)	234 (91.1)	
	1	16 (8.3)	7 (10.8)	23 (8.9)	0.7313783
event-free survival second_malignancy	0	187 (97.4)	64 (98.5)	251 (97.7)	
	1	5 (2.6)	1 (1.5)	6 (2.3)	0.9867236

**Table 2:** Clinical characteristics from MB patients with processed RNA-sequencing samples in validation cohort (E-MTAB-10767): For respective epidemiological parameters, numbers and percentage of patients were described in Total column for qualitative parameter and means/standard deviations for quantitative parameters. Cohort stratification for low and high subtypes was performed on ferroptosis expression score threshold determined during this study. *p*-values test the difference between low- and high-ferroptosis patient subtypes by chi-square test for qualitative parameters and *t*-test for quantitative parameters.

Variable	Level	Low (n = 214)	High (n = 117)	Total (n = 331)	*p*-Value
developmental stage	adult	6 (2.8)	3 (2.6)	9 (2.7)	
	infant	23 (10.7)	21 (17.9)	44 (13.3)	
	child	167 (78.0)	79 (67.5)	246 (74.3)	
	not available	18 (8.4)	14 (12.0)	32 (9.7)	0.16596
gender	male	132 (61.7)	62 (53.0)	194 (58.6)	
	female	60 (28.0)	41 (35.0)	101 (30.5)	
	not available	22 (10.3)	14 (12.0)	36 (10.9)	0.30286
subgroup	Grp4	127 (59.3)	20 (17.1)	147 (44.4)	
	SHH	33 (15.4)	34 (29.1)	67 (20.2)	
	MB-NOS	6 (2.8)	4 (3.4)	10 (3.0)	
	Grp3	23 (10.7)	40 (34.2)	63 (19.0)	
	WNT	17 (7.9)	14 (12.0)	31 (9.4)	
	Grp3/Grp4	8 (3.7)	5 (4.3)	13 (3.9)	<1 × 10^−4^
Overall survival time (years)	mean (sd)	6.2 (10)	3.8 (3.8)	5.5 (8.8)	0.08842

**Table 3:** Cross-validated ferroptosis signature associated with the prognosis of medulloblastoma.

Identifiers	Prognosis	Negative Log10 *p*-Values	Cox Beta Coefficients
CCT3	unfavorable	4.404	1.368
SNX5	unfavorable	2.318	1.356
SQOR	unfavorable	2.559	1.332
G3BP1	unfavorable	2.169	1.178
CARS1	unfavorable	1.991	1.170
SLC39A14	unfavorable	2.682	1.163
FAM98A	unfavorable	1.656	0.999
FXR1	unfavorable	1.577	0.979
TFAP2C	unfavorable	3.549	0.968
ATF4	unfavorable	2.119	0.956
TXN	unfavorable	3.291	0.941
MTDH	unfavorable	1.580	0.910
AHCY	unfavorable	3.129	0.788
TIMM9	unfavorable	1.475	0.736
CAV1	unfavorable	2.258	0.723
KIF20A	unfavorable	1.538	0.718
PRDX4	unfavorable	2.042	0.704
STC1	unfavorable	1.840	0.589
IL6	unfavorable	1.535	0.577
CBS	unfavorable	1.870	0.553
PHGDH	unfavorable	1.589	0.483
PRR5	favorable	1.513	−0.540
CEMIP	favorable	2.509	−0.590
ADAM23	favorable	1.484	−0.615
CUL9	favorable	1.493	−0.711
TSC1	favorable	1.802	−0.711
HPX	favorable	1.686	−0.716
TCF4	favorable	2.218	−0.755
VAMP2	favorable	2.224	−0.758
TFR2	favorable	1.683	−0.798
PCDHB14	favorable	1.902	−0.812
CAPRIN2	favorable	1.952	−0.852
MAP1LC3A	favorable	2.195	−0.861
CDO1	favorable	3.232	−0.872
BEX1	favorable	3.414	−0.908
PPARA	favorable	1.556	−0.924
SOX15	favorable	4.387	−0.992
MCF2L	favorable	4.384	−1.045
SMG9	favorable	1.385	−1.159
CLOCK	favorable	2.008	−1.209
PARP6	favorable	2.469	−1.363
TRPV1	favorable	2.961	−1.466
USF2	favorable	2.037	−1.474
COQ10A	favorable	3.324	−1.688
USP11	favorable	5.137	−2.180

**Table 4:** Summary table of the multivariable overall survival model for medulloblastoma prognosis in PBTA cohort.

Term	Hazard Ratios	CI95-Low	CI95-High	*p*-Values
score.cat (high)	5.836	3.312	10.284	1.04 × 10^−9^
older.15yo (high)	0.505	0.210	1.213	1.26 × 10^−1^
gender (Male)	0.919	0.571	1.480	7.30 × 10^−1^
groups (group.4)	0.994	0.526	1.877	9.84 × 10^−1^
groups (SHH-activated)	1.260	0.719	2.209	4.20 × 10^−1^

## Data Availability

Scripts computed during this study are available at the following web address: https://github.com/cdesterke/scriptsForFerroptosisMedulloblastoma (accessed on 7 December 2024).

## Supplementary Materials

The following supporting information can be downloaded at: https://www.mdpi.com/article/10.3390/antiox14010096/s1, Supplementary Figures: Supplementary Figure S1. RNA-sequencing preprocess of training cohort of Medulloblastoma tumors; Supplementary Figure S2. RNA-sequencing preprocess of validation cohort of Medulloblastoma tumors: RNA-sequencing preprocess of training cohort of Medulloblastoma tumors; Supplementary Figure S3. Preprocess for gene-regulated network (WGCNA) for ferroptosis related genes in PBTA RNA-sequencing cohort; Supplementary Figure S4. Accessory data for the construction of the multivariable overall survival model on PBTA cohort with exclusion of WNT-subgroup; Supplementary Tables: Supplementary Table S1. Univariate overall survival Cox analyses performed on expression of ferroptosis-related genes in training cohort; Supplementary Table S2. Univariate overall survival Cox analyses performed on expression of ferroptosis-related genes in validation cohort.
