# Supplementary material for: Ferroptosis Transcriptional Regulation and Prognostic Impact in Medulloblastoma Subtypes Revealed by RNA-Seq
Source: Antioxidants (Basel). 2025 Jan 15;14(1):96. doi: 10.3390/antiox14010096 (PMC11761645; doi:10.3390/antiox14010096)
Supplement: Supplementary file 1 [file antioxidants-14-00096-s001.zip › antioxidants-3393733-supplementary.pdf]

antioxidants

Supplementary material

Ferroptosis Transcriptional Regulation and Prognostic Impact in Medulloblastoma Subtypes Revealed by RNA-Seq

Christophe Desterke [1], Yuanji Fu [2], Jenny Bonifacio-Mundaca [3], Claudia Monge [4], Pascal Pineau [4], Jorge Mata-Garrido [4,*,†] and Raquel Francés [5,*,†]

1 INSERM UMRS-1310, Faculté de Médecine du Kremlin Bicêtre, Université Paris-Saclay, F-94270 Le Kremlin-Bicêtre, France; christophe.desterke@inserm.fr
2 INSERM, CNRS, Institut Necker Enfants Malades, Université Paris Cité, F-75015 Paris, France; yuanji.fu@inserm.fr
3 National Tumor Bank, Department of Pathology, National Institute of Neoplastic Diseases, Surquillo 15038, Peru; jenny.bonifacio@upch.pe
4 Unité Organisation Nucléaire et Oncogenèse, Institut Pasteur, Université Paris Cité, INSERM U993, F-75015 Paris, France; claudia.monge@pasteur.fr (C.M.); pascal.pineau@pasteur.fr (P.P.)
5 Energy & Memory, Brain Plasticity Unit, CNRS, ESPCI Paris, PSL Research University, F-75006 Paris, France
* Correspondence: jorge.mata-garrido@pasteur.fr (J.M.-G.); raquel.frances@espci.fr (R.F.)
† These authors contributed equally to this work.

 https://doi.org/10.3390/antiox14010096

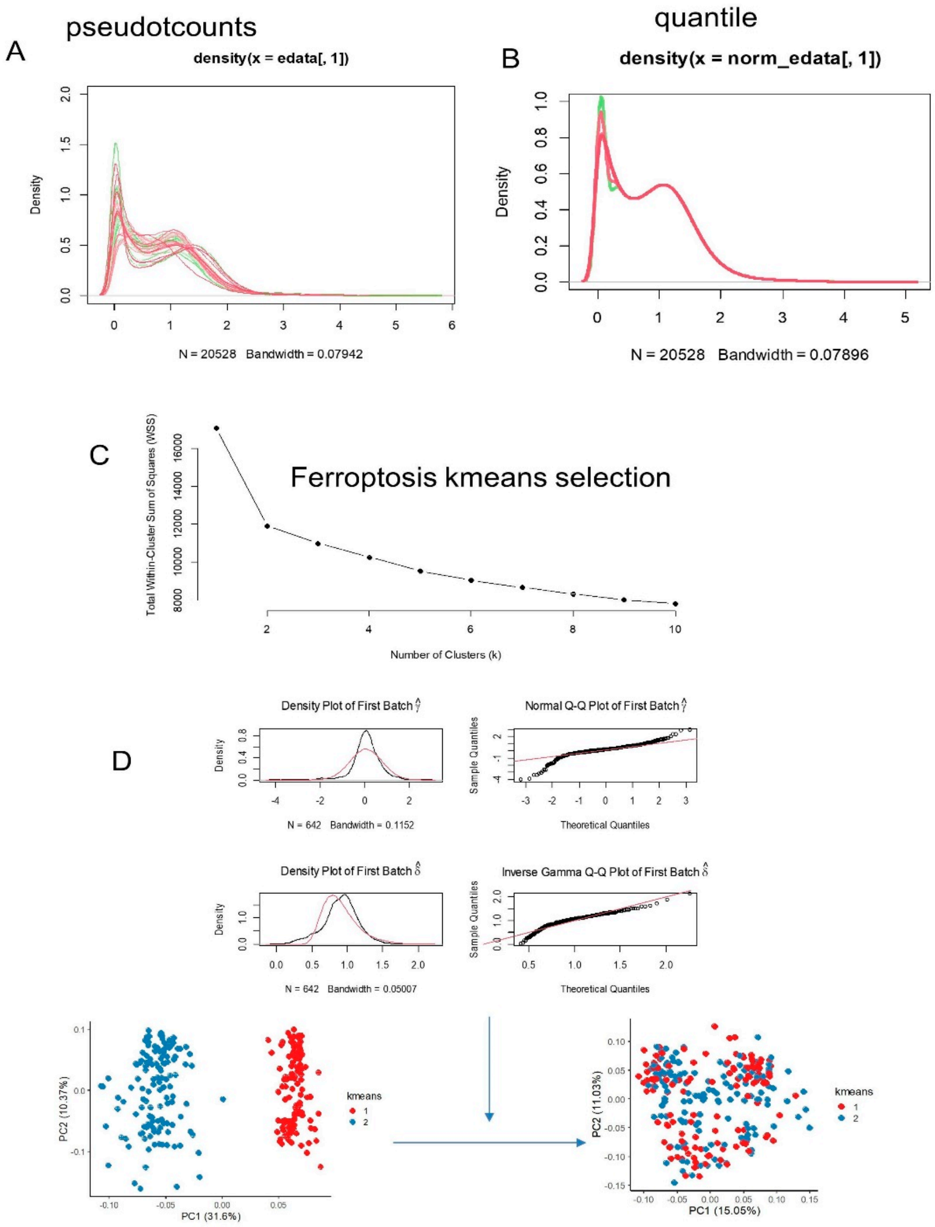

Supplementary Figure 1. RNA-sequencing preprocessing of the training cohort of medulloblastoma tumors. (A) Density plot of filtered RNA-seq pseudocounts. (B) Density plot of quantile-normalized pseudocounts. (C) Cluster detection using the k-means algorithm based on ferroptosis-related gene expression. (D) Combat batch-corrected normalization of ferroptosis-related gene quantification in the training cohort.

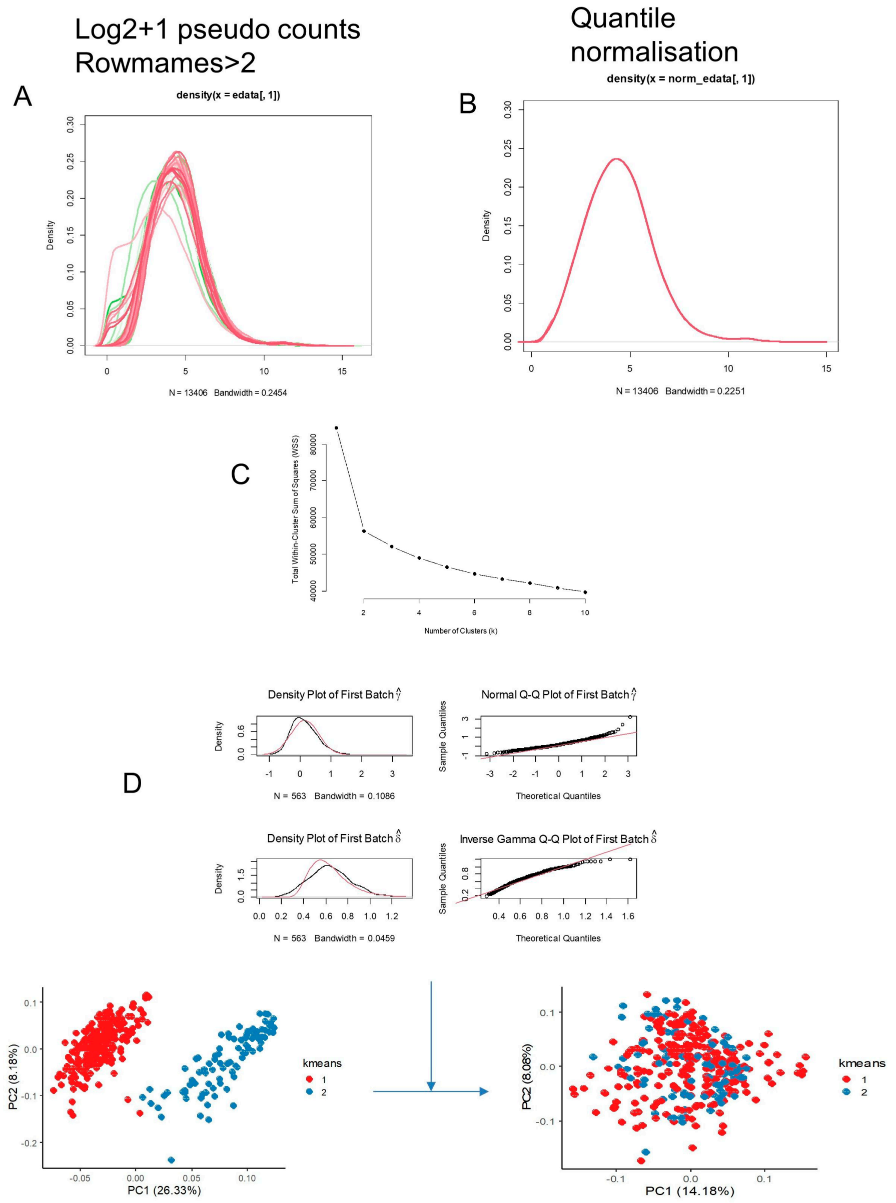

Supplementary Figure 2. RNA-sequencing preprocessing of the validation cohort of medulloblastoma tumors. (A) Density plot of filtered RNA-seq pseudocounts. (B) Density plot of quantile-normalized pseudocounts. (C) Cluster detection using the k-means algorithm based on ferroptosis-related gene expression. (D) Combat batch-corrected normalization of ferroptosis-related gene quantification in the validation cohort.

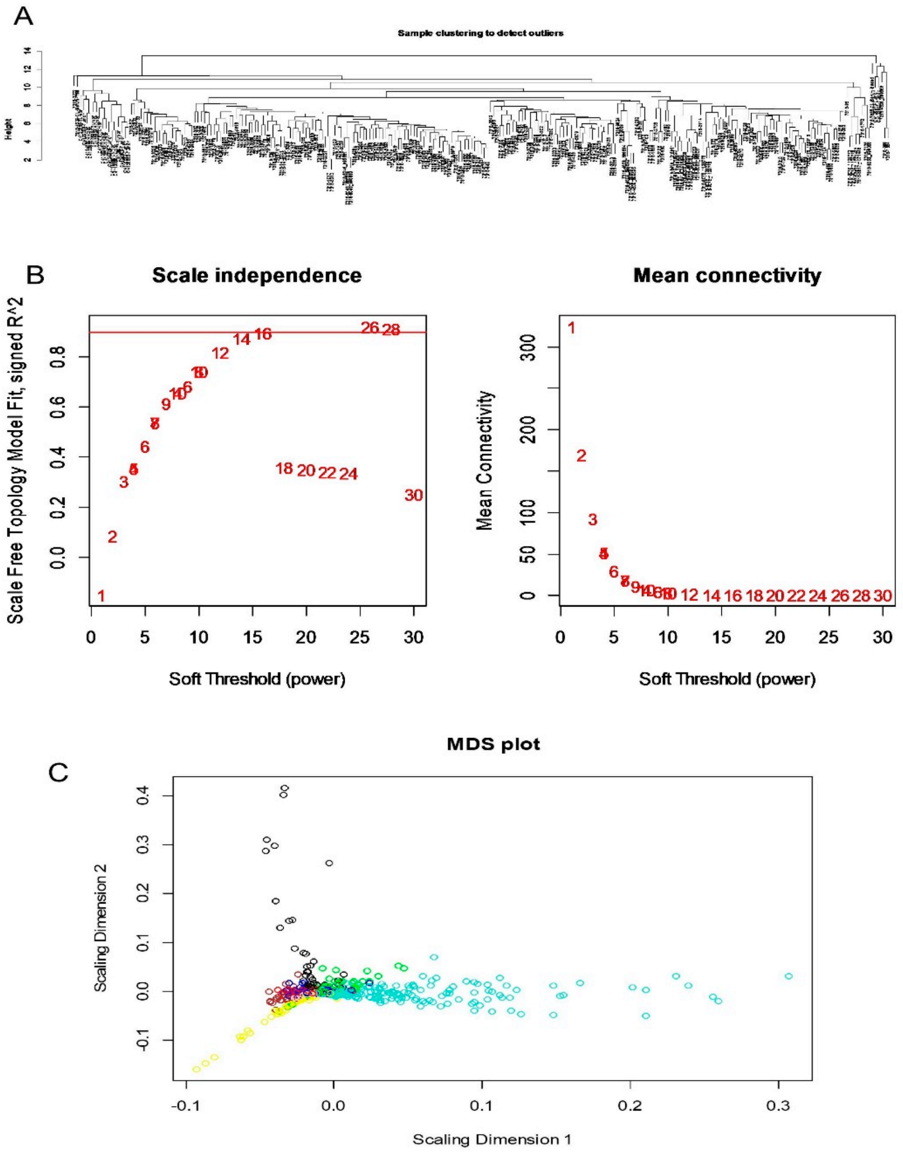

Supplemental Figure 3. Preprocessing for gene regulatory network (WGCNA) analysis of ferroptosis-related genes in the PBTA RNA-sequencing cohort. (A) Clustering tree of samples that passed outlier filtration before the construction of the network with ferroptosis-related genes. (B) Soft power selection for WGCNA analysis based on the expression of ferroptosis-related genes in the PBTA RNA-seq cohort. (C) Dimensionality reduction plot for the gene regulatory modules identified during WGCNA analysis.

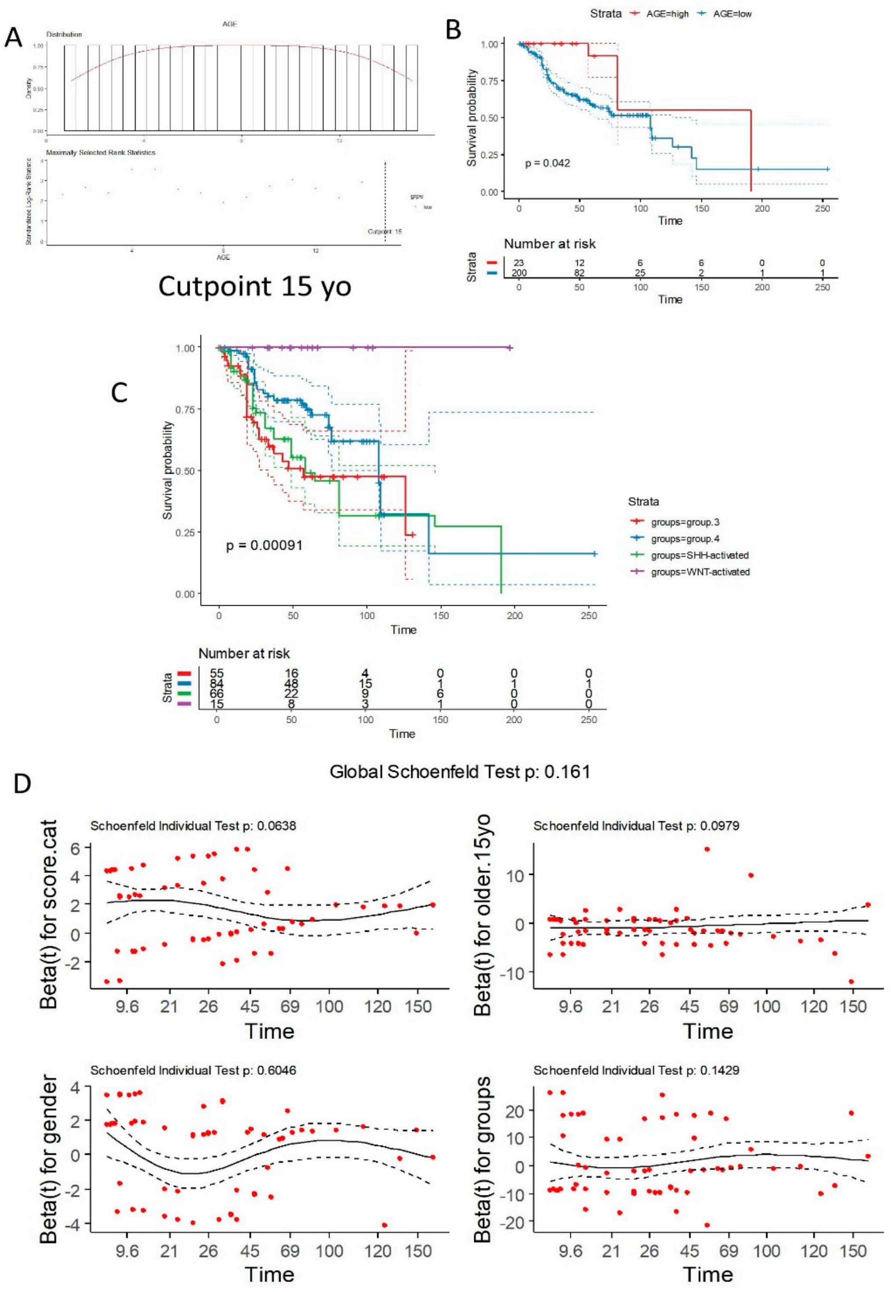

Supplemental Figure 4. Additional data for the construction of the multivariable overall survival model in the PBTA cohort with exclusion of the WNT subgroup: (A) Detection of the optimal cutoff for the age at diagnosis parameter based on log-rank residuals from overall survival analysis. (B) Kaplan-Meier survival curve and log-rank test p-value for age at diagnosis, stratified by the 15-year cutoff. (C) Kaplan-Meier survival curve and log-rank test for molecular subgroup stratification. (D) Global and individual Schoenfeld tests for the multivariable overall survival model.

Supplemental Table 1. Univariate overall survival Cox analyses performed on expression of ferroptosis related genes in training cohort.

identifiers	coef.beta	Hazard ratios	p-values	significance	prognosis
PLA2G6	-3.205	0.041	1.34E-08	YES	favorable
USP11	-3.180	0.042	9.12E-08	YES	favorable
H19	0.840	2.317	2.34E-07	YES	unfavorable
CDKN1A	1.491	4.441	2.73E-07	YES	unfavorable
SIRT6	-3.408	0.033	3.28E-07	YES	favorable
MCF2L	-1.573	0.207	1.11E-06	YES	favorable
SOX15	-1.422	0.241	1.23E-06	YES	favorable
ARMC5	-2.688	0.068	2.03E-06	YES	favorable
PARP3	-2.165	0.115	2.41E-06	YES	favorable
LPIN1	-2.872	0.057	3.07E-06	YES	favorable
BEX1	-1.186	0.305	7.69E-06	YES	favorable
GDF15	1.713	5.544	1.01E-05	YES	unfavorable
SCARA5	1.402	4.064	2.84E-05	YES	unfavorable
CDO1	-1.288	0.276	3.85E-05	YES	favorable
CERK	-1.786	0.168	4.01E-05	YES	favorable
TRPV1	-2.334	0.097	4.13E-05	YES	favorable
ENPP2	0.855	2.352	6.60E-05	YES	unfavorable
PARP8	-1.474	0.229	7.04E-05	YES	favorable
ELOVL5	2.302	9.996	8.77E-05	YES	unfavorable
PARP10	-1.941	0.144	1.12E-04	YES	favorable
COQ10A	-2.406	0.090	1.38E-04	YES	favorable
SCD	0.992	2.696	1.52E-04	YES	unfavorable
PLIN2	1.169	3.219	1.67E-04	YES	unfavorable
NDRG1	1.010	2.746	1.79E-04	YES	unfavorable
ENO1	1.228	3.413	1.82E-04	YES	unfavorable
PIM1	1.342	3.828	1.86E-04	YES	unfavorable
ADAMTS13	-1.860	0.156	2.05E-04	YES	favorable
ZFAS1	0.849	2.338	2.26E-04	YES	unfavorable
CS	2.348	10.465	3.40E-04	YES	unfavorable
MAP3K14	-1.929	0.145	3.55E-04	YES	favorable
MEN1	-2.565	0.077	3.79E-04	YES	favorable
BAP1	-2.380	0.093	5.28E-04	YES	favorable
FABP4	0.608	1.837	5.38E-04	YES	unfavorable
SRC	-1.215	0.297	6.35E-04	YES	favorable
TFAP2C	1.225	3.405	6.56E-04	YES	unfavorable
KDM6B	-1.119	0.327	6.81E-04	YES	favorable
CAV1	0.888	2.430	8.32E-04	YES	unfavorable
SNX5	1.679	5.359	8.44E-04	YES	unfavorable
ADORA2B	1.489	4.434	8.54E-04	YES	unfavorable
PARP6	-1.945	0.143	8.69E-04	YES	favorable
TRIM59	1.150	3.159	8.69E-04	YES	unfavorable

SQOR	1.571	4.813	9.18E-04	YES	unfavorable
FSCN1	-1.000	0.368	9.50E-04	YES	favorable
ULK1	-1.185	0.306	9.70E-04	YES	favorable
KAT5	-2.531	0.080	9.78E-04	YES	favorable
SLC39A14	1.626	5.083	1.06E-03	YES	unfavorable
ZSCAN25	-1.629	0.196	1.20E-03	YES	favorable
MAP1LC3A	-1.199	0.302	1.47E-03	YES	favorable
GABARAPL1	0.791	2.206	1.56E-03	YES	unfavorable
SENP1	-1.940	0.144	1.88E-03	YES	favorable
CEMIP	-0.674	0.510	2.02E-03	YES	favorable
TXN	0.772	2.163	2.04E-03	YES	unfavorable
HULC	-1.601	0.202	2.08E-03	YES	favorable
PVT1	0.553	1.738	2.41E-03	YES	unfavorable
LRP8	-0.823	0.439	2.61E-03	YES	favorable
ME1	1.443	4.233	2.77E-03	YES	unfavorable
POR	-1.459	0.233	3.32E-03	YES	favorable
CREB1	-1.260	0.284	3.74E-03	YES	favorable
MLST8	-1.791	0.167	4.11E-03	YES	favorable
PEX14	-2.183	0.113	4.19E-03	YES	favorable
PRDX6	0.961	2.614	4.24E-03	YES	unfavorable
PDK4	0.773	2.166	4.26E-03	YES	unfavorable
BRPF1	-2.003	0.135	4.50E-03	YES	favorable
SIRT3	-1.934	0.145	4.55E-03	YES	favorable
VDAC2	1.220	3.388	4.98E-03	YES	unfavorable
NSUN5	-1.799	0.166	5.69E-03	YES	favorable
YY2	-3.076	0.046	5.70E-03	YES	favorable
PCDHB14	-1.132	0.322	7.06E-03	YES	favorable
STK11	-1.779	0.169	7.23E-03	YES	favorable
POU2F2	-1.103	0.332	7.25E-03	YES	favorable
MPC1	0.943	2.569	7.40E-03	YES	unfavorable
AQP3	-0.768	0.464	7.79E-03	YES	favorable
CTSB	1.227	3.411	8.06E-03	YES	unfavorable
MLLT1	-1.188	0.305	8.42E-03	YES	favorable
CHMP6	-0.934	0.393	8.88E-03	YES	favorable
NUAK2	-1.346	0.260	9.02E-03	YES	favorable
USP22	-1.145	0.318	9.45E-03	YES	favorable
DDIT4	0.693	1.999	9.70E-03	YES	unfavorable
LONP1	-1.696	0.183	9.90E-03	YES	favorable
LTF	1.274	3.575	9.99E-03	YES	unfavorable
S100A8	0.429	1.536	1.04E-02	YES	unfavorable
BRD3	-0.901	0.406	1.08E-02	YES	favorable
KDM4A	-1.819	0.162	1.18E-02	YES	favorable
PARP16	-2.073	0.126	1.18E-02	YES	favorable
MIB2	-0.662	0.516	1.27E-02	YES	favorable

ASAP2	-1.248	0.287	1.29E-02	YES	favorable
A2M-AS1	-1.476	0.228	1.31E-02	YES	favorable
RPTOR	-1.008	0.365	1.32E-02	YES	favorable
TFR2	-1.104	0.331	1.36E-02	YES	favorable
USF2	-1.701	0.183	1.41E-02	YES	favorable
SOCS2	-0.658	0.518	1.46E-02	YES	favorable
SLC7A11	1.053	2.865	1.52E-02	YES	unfavorable
FBXW7	-1.030	0.357	1.53E-02	YES	favorable
LAMP2	1.213	3.364	1.57E-02	YES	unfavorable
ATF4	0.759	2.136	1.57E-02	YES	unfavorable
FBXO31	-1.026	0.358	1.59E-02	YES	favorable
MCU	1.258	3.519	1.61E-02	YES	unfavorable
PHGDH	0.501	1.651	1.64E-02	YES	unfavorable
ACO1	1.563	4.772	1.68E-02	YES	unfavorable
BCAT1	0.563	1.756	1.70E-02	YES	unfavorable
RPL8	0.749	2.115	1.71E-02	YES	unfavorable
SUFU	-1.185	0.306	1.73E-02	YES	favorable
SIRT7	-1.409	0.244	1.75E-02	YES	favorable
PPARA	-1.130	0.323	1.79E-02	YES	favorable
NUP62	-1.924	0.146	1.80E-02	YES	favorable
G3BP1	1.340	3.819	1.80E-02	YES	unfavorable
EMC2	1.045	2.845	1.86E-02	YES	unfavorable
ANO6	1.016	2.762	1.86E-02	YES	unfavorable
FOXO3	1.392	4.023	1.91E-02	YES	unfavorable
PRR5	-0.700	0.497	1.93E-02	YES	favorable
HPCAL1	-0.612	0.542	2.01E-02	YES	favorable
EIF2AK2	-1.552	0.212	2.03E-02	YES	favorable
MDM4	-0.780	0.458	2.08E-02	YES	favorable
CARS1	1.515	4.548	2.09E-02	YES	unfavorable
PRDX4	0.699	2.012	2.11E-02	YES	unfavorable
SNAI2	0.821	2.272	2.19E-02	YES	unfavorable
CCT3	0.906	2.474	2.21E-02	YES	unfavorable
PEX10	-1.479	0.228	2.27E-02	YES	favorable
METTL16	-1.617	0.199	2.36E-02	YES	favorable
SELENOP	0.468	1.597	2.38E-02	YES	unfavorable
PEX12	-1.308	0.270	2.39E-02	YES	favorable
ANGPTL4	0.650	1.915	2.41E-02	YES	unfavorable
DPEP1	-0.792	0.453	2.42E-02	YES	favorable
SREBF2	1.376	3.957	2.42E-02	YES	unfavorable
TSC1	-0.876	0.417	2.43E-02	YES	favorable
CBS	0.548	1.729	2.46E-02	YES	unfavorable
ATF3	0.630	1.877	2.47E-02	YES	unfavorable
SMAD7	-0.929	0.395	2.47E-02	YES	favorable
BDNF-AS	-1.419	0.242	2.53E-02	YES	favorable

MTTP	-2.354	0.095	2.56E-02	YES	favorable
KMT2D	-0.889	0.411	2.60E-02	YES	favorable
CLOCK	-1.126	0.324	2.62E-02	YES	favorable
TBK1	-1.383	0.251	2.64E-02	YES	favorable
TAFAZZIN	-1.149	0.317	2.65E-02	YES	favorable
RARRES2	0.524	1.689	2.65E-02	YES	unfavorable
VAMP2	-0.762	0.467	2.68E-02	YES	favorable
IL6	0.633	1.883	2.74E-02	YES	unfavorable
TRPM7	-1.133	0.322	2.79E-02	YES	favorable
TIMM9	0.787	2.198	2.82E-02	YES	unfavorable
TCF4	-0.745	0.475	3.04E-02	YES	favorable
HDDC3	-1.528	0.217	3.07E-02	YES	favorable
MTCH1	1.394	4.030	3.16E-02	YES	unfavorable
DECR1	0.745	2.107	3.21E-02	YES	unfavorable
NF2	-1.130	0.323	3.25E-02	YES	favorable
SLC25A11	-1.313	0.269	3.28E-02	YES	favorable
DUOX1	-0.715	0.489	3.30E-02	YES	favorable
METTL14	-0.889	0.411	3.32E-02	YES	favorable
ERN1	-0.874	0.417	3.34E-02	YES	favorable
HUWE1	-1.339	0.262	3.41E-02	YES	favorable
STC1	0.514	1.671	3.48E-02	YES	unfavorable
NCOA4	1.240	3.454	3.52E-02	YES	unfavorable
IREB2	-1.155	0.315	3.55E-02	YES	favorable
PARP12	-0.805	0.447	3.59E-02	YES	favorable
COX4I2	0.588	1.801	3.59E-02	YES	unfavorable
YY1AP1	-1.520	0.219	3.64E-02	YES	favorable
PSEN2	-1.049	0.350	3.65E-02	YES	favorable
ASMTL-AS1	-0.948	0.388	3.66E-02	YES	favorable
ADIPOQ	0.719	2.053	3.76E-02	YES	unfavorable
ACSL4	-0.932	0.394	3.76E-02	YES	favorable
ADAM23	-0.715	0.489	3.76E-02	YES	favorable
HPX	-1.033	0.356	3.80E-02	YES	favorable
PPARG	0.898	2.454	3.81E-02	YES	unfavorable
HILPDA	0.789	2.201	3.88E-02	YES	unfavorable
SLC47A1	0.907	2.476	3.91E-02	YES	unfavorable
KIF20A	0.800	2.225	3.94E-02	YES	unfavorable
FAM98A	1.056	2.874	4.01E-02	YES	unfavorable
SMG9	-1.420	0.242	4.06E-02	YES	favorable
GRIA3	1.027	2.792	4.07E-02	YES	unfavorable
H3C7	-0.207	0.813	4.11E-02	YES	favorable
MTHFR	-0.842	0.431	4.23E-02	YES	favorable
SMPD1	-1.184	0.306	4.36E-02	YES	favorable
RBMS1	0.944	2.571	4.37E-02	YES	unfavorable
PEDS1	1.413	4.107	4.37E-02	YES	unfavorable

CAPRIN2	-0.836	0.434	4.48E-02	YES	favorable
CAVIN1	0.653	1.921	4.49E-02	YES	unfavorable
PCAT1	0.745	2.107	4.62E-02	YES	unfavorable
MTDH	1.094	2.985	4.64E-02	YES	unfavorable
MIR9-3HG	-0.584	0.558	4.79E-02	YES	favorable
FXR1	0.740	2.096	4.82E-02	YES	unfavorable
AHCY	0.680	1.974	4.82E-02	YES	unfavorable
CUL9	-0.885	0.413	4.93E-02	YES	favorable
GOT1	0.761	2.140	4.99E-02	YES	unfavorable
SDHA	1.093	2.982	5.12E-02	no	unfavorable
ATM	-0.949	0.387	5.18E-02	no	favorable
WIPI1	0.863	2.371	5.21E-02	no	unfavorable
SOCS1	-0.705	0.494	5.29E-02	no	favorable
RND1	-0.560	0.571	5.33E-02	no	favorable
BRCA1	-0.798	0.450	5.36E-02	no	favorable
USP35	-0.857	0.425	5.43E-02	no	favorable
KDM3B	-1.714	0.180	5.52E-02	no	favorable
SUV39H1	-1.488	0.226	5.54E-02	no	favorable
SAT1	0.738	2.091	5.65E-02	no	unfavorable
KDM5A	-0.765	0.465	5.76E-02	no	favorable
NPC1	1.283	3.607	5.83E-02	no	unfavorable
KMT2B	-0.982	0.375	5.85E-02	no	favorable
BRD4	-0.692	0.500	5.88E-02	no	favorable
UBIAD1	-1.238	0.290	6.03E-02	no	favorable
GJA1	0.478	1.613	6.17E-02	no	unfavorable
SDHB	0.991	2.693	6.36E-02	no	unfavorable
ENO3	-0.482	0.618	6.38E-02	no	favorable
H3C1	-0.216	0.806	6.44E-02	no	favorable
H3C8	-0.184	0.832	6.61E-02	no	favorable
MYC	0.309	1.362	6.73E-02	no	unfavorable
CD74	-0.552	0.576	6.80E-02	no	favorable
AQP5	-0.442	0.642	6.90E-02	no	favorable
WIPI2	-1.180	0.307	7.01E-02	no	favorable
TOR2A	-1.099	0.333	7.12E-02	no	favorable
AIFM2	-1.101	0.332	7.14E-02	no	favorable
MIB1	-0.956	0.384	7.17E-02	no	favorable
BECN1	-1.097	0.334	7.18E-02	no	favorable
C19orf12	-1.487	0.226	7.22E-02	no	favorable
SIAH2	0.618	1.855	7.52E-02	no	unfavorable
MTOR	-1.114	0.328	7.68E-02	no	favorable
HSPA8	0.576	1.778	7.92E-02	no	unfavorable
FTL	0.522	1.685	8.02E-02	no	unfavorable
MXRA8	-0.430	0.651	8.03E-02	no	favorable
LIG3	-0.725	0.484	8.33E-02	no	favorable

AKR1C3	0.574	1.775	8.47E-02	no	unfavorable
SDHD	0.447	1.564	8.53E-02	no	unfavorable
H3C6	-0.206	0.814	8.63E-02	no	favorable
BMP4	-0.489	0.613	8.67E-02	no	favorable
SPATA2	-1.158	0.314	8.87E-02	no	favorable
FNDC5	-0.600	0.549	8.97E-02	no	favorable
H3C3	-0.187	0.829	9.02E-02	no	favorable
CREB5	0.513	1.671	9.22E-02	no	unfavorable
MITF	0.901	2.461	9.31E-02	no	unfavorable
RAPGEF3	-0.832	0.435	9.52E-02	no	favorable
MGST1	0.350	1.419	9.79E-02	no	unfavorable
HERPUD1	0.867	2.380	9.83E-02	no	unfavorable
CDC27	-1.067	0.344	9.91E-02	no	favorable
NAT10	1.160	3.190	9.96E-02	no	unfavorable
KLHDC3	1.020	2.773	1.00E-01	no	unfavorable
PROM2	-0.848	0.428	1.01E-01	no	favorable
NOTCH3	0.457	1.580	1.01E-01	no	unfavorable
FOXO4	0.576	1.778	1.03E-01	no	unfavorable
ALG3	0.938	2.555	1.04E-01	no	unfavorable
H3C12	-0.175	0.839	1.05E-01	no	favorable
PPARD	-0.940	0.391	1.05E-01	no	favorable
CP	0.400	1.491	1.07E-01	no	unfavorable
FTH1	0.482	1.620	1.07E-01	no	unfavorable
TULP1	-0.232	0.793	1.08E-01	no	favorable
AQP11	-1.260	0.284	1.08E-01	no	favorable
GGT1	-0.838	0.433	1.11E-01	no	favorable
ULK2	-0.719	0.487	1.11E-01	no	favorable
MALAT1	-0.270	0.763	1.12E-01	no	favorable
EZH2	-0.578	0.561	1.12E-01	no	favorable
RBM24	-0.365	0.694	1.12E-01	no	favorable
PTPN18	-0.802	0.448	1.15E-01	no	favorable
NQO1	0.495	1.640	1.15E-01	no	unfavorable
KRT18	-0.495	0.610	1.18E-01	no	favorable
INTS2	-0.784	0.457	1.19E-01	no	favorable
CD82	-0.509	0.601	1.20E-01	no	favorable
EMP1	0.420	1.521	1.20E-01	no	unfavorable
HSF1	0.810	2.249	1.21E-01	no	unfavorable
TFAP2A	-0.453	0.636	1.21E-01	no	favorable
H3C13	-0.180	0.835	1.23E-01	no	favorable
GRSF1	0.866	2.377	1.23E-01	no	unfavorable
TXNRD1	0.841	2.318	1.23E-01	no	unfavorable
ETHE1	0.903	2.468	1.24E-01	no	unfavorable
MAPK8	-0.646	0.524	1.24E-01	no	favorable
HMOX1	0.413	1.512	1.25E-01	no	unfavorable

USP7	-1.205	0.300	1.28E-01	no	favorable
GCLC	0.768	2.156	1.28E-01	no	unfavorable
PAQR3	-0.748	0.473	1.29E-01	no	favorable
COMMD10	0.662	1.939	1.32E-01	no	unfavorable
H3C11	-0.138	0.871	1.33E-01	no	favorable
NEDD4	0.819	2.269	1.35E-01	no	unfavorable
ARHGEF26-AS1	0.698	2.009	1.36E-01	no	unfavorable
FANCD2	-0.614	0.541	1.36E-01	no	favorable
ABHD12	0.904	2.468	1.36E-01	no	unfavorable
TIMP1	0.328	1.388	1.38E-01	no	unfavorable
OMA1	-0.684	0.505	1.38E-01	no	favorable
ATG16L1	-1.184	0.306	1.38E-01	no	favorable
HDLBP	0.734	2.084	1.38E-01	no	unfavorable
KAT6B	-0.546	0.579	1.39E-01	no	favorable
GSK3B	-0.719	0.487	1.42E-01	no	favorable
AURKA	0.639	1.894	1.43E-01	no	unfavorable
PANX2	-0.556	0.573	1.45E-01	no	favorable
CAMKK2	0.517	1.677	1.46E-01	no	unfavorable
H3C10	-0.169	0.845	1.46E-01	no	favorable
H3C4	-0.150	0.860	1.48E-01	no	favorable
MAP3K11	-0.674	0.510	1.48E-01	no	favorable
DKK1	-0.483	0.617	1.49E-01	no	favorable
LINC00472	-0.885	0.413	1.51E-01	no	favorable
SPHK1	0.313	1.367	1.51E-01	no	unfavorable
SLC25A37	0.474	1.607	1.53E-01	no	unfavorable
SSBP1	0.463	1.589	1.54E-01	no	unfavorable
ARNTL	-0.520	0.595	1.56E-01	no	favorable
NUPR1	0.269	1.308	1.56E-01	no	unfavorable
ATP5MC3	0.578	1.782	1.56E-01	no	unfavorable
APOE	-0.366	0.693	1.56E-01	no	favorable
TIGAR	-0.273	0.761	1.58E-01	no	favorable
APP	-0.343	0.710	1.64E-01	no	favorable
PARK7	0.466	1.594	1.66E-01	no	unfavorable
NRAS	0.689	1.991	1.68E-01	no	unfavorable
H3C2	-0.128	0.880	1.71E-01	no	favorable
PEBP1	0.552	1.737	1.73E-01	no	unfavorable
PER1	-0.476	0.621	1.74E-01	no	favorable
ATF2	-0.992	0.371	1.75E-01	no	favorable
CHAC1	0.767	2.152	1.76E-01	no	unfavorable
MT1G	0.242	1.274	1.81E-01	no	unfavorable
ACADSB	-0.592	0.553	1.83E-01	no	favorable
TNFAIP1	-0.622	0.537	1.83E-01	no	favorable
LINC01833	-0.375	0.688	1.85E-01	no	favorable
AQP4	-0.249	0.780	1.86E-01	no	favorable

JUP	-0.368	0.692	1.87E-01	no	favorable
ALDH3A2	-0.665	0.514	1.92E-01	no	favorable
SIRT2	0.572	1.772	1.92E-01	no	unfavorable
PRDX2	-0.713	0.490	1.92E-01	no	favorable
HRH1	0.833	2.301	1.93E-01	no	unfavorable
PARP11	-0.738	0.478	1.97E-01	no	favorable
CHMP1A	-0.860	0.423	1.97E-01	no	favorable
AEBP2	-0.888	0.412	2.01E-01	no	favorable
CCDC6	1.176	3.243	2.02E-01	no	unfavorable
NFS1	-0.922	0.398	2.02E-01	no	favorable
PCBP2	0.736	2.089	2.03E-01	no	unfavorable
NOS2	0.638	1.892	2.05E-01	no	unfavorable
GLS2	-0.536	0.585	2.06E-01	no	favorable
CREB3	-0.868	0.420	2.07E-01	no	favorable
STING1	0.508	1.662	2.08E-01	no	unfavorable
SLC39A7	0.623	1.865	2.09E-01	no	unfavorable
ITGB3	0.612	1.844	2.10E-01	no	unfavorable
ADORA1	-0.361	0.697	2.11E-01	no	favorable
H3C14	-0.144	0.865	2.12E-01	no	favorable
CLTRN	-0.887	0.412	2.12E-01	no	favorable
NT5DC2	-0.625	0.535	2.13E-01	no	favorable
SHARPIN	-0.798	0.450	2.14E-01	no	favorable
LIFR	0.497	1.644	2.16E-01	no	unfavorable
SLC38A1	-0.248	0.780	2.19E-01	no	favorable
PDIA2	0.252	1.287	2.19E-01	no	unfavorable
HMGCR	0.555	1.742	2.19E-01	no	unfavorable
PML	-0.676	0.509	2.20E-01	no	favorable
RHEBP1	-0.636	0.529	2.25E-01	no	favorable
TRIM26	-0.673	0.510	2.30E-01	no	favorable
AGPAT3	-0.699	0.497	2.31E-01	no	favorable
FGFR4	0.396	1.486	2.35E-01	no	unfavorable
PHF21A	-0.582	0.559	2.36E-01	no	favorable
CDCA3	-0.390	0.677	2.37E-01	no	favorable
PCSK9	-0.264	0.768	2.39E-01	no	favorable
NTN1	-0.246	0.782	2.40E-01	no	favorable
KLF2	-0.319	0.727	2.45E-01	no	favorable
ASAH2	-0.760	0.468	2.52E-01	no	favorable
DCAF7	-0.586	0.556	2.53E-01	no	favorable
BRD2	0.768	2.156	2.53E-01	no	unfavorable
PSEN1	-0.803	0.448	2.57E-01	no	favorable
GPX7	0.380	1.462	2.57E-01	no	unfavorable
JUND	-0.249	0.779	2.58E-01	no	favorable
CHP1	0.743	2.103	2.58E-01	no	unfavorable
GALNT14	-0.189	0.828	2.59E-01	no	favorable

SDHC	0.299	1.349	2.60E-01	no	unfavorable
PER2	-0.522	0.593	2.61E-01	no	favorable
FTO	-0.660	0.517	2.62E-01	no	favorable
YTHDC2	-0.585	0.557	2.67E-01	no	favorable
PRKCA	0.413	1.512	2.68E-01	no	unfavorable
HLF	-0.324	0.723	2.68E-01	no	favorable
RELA	-0.749	0.473	2.70E-01	no	favorable
PEX3	-0.595	0.551	2.71E-01	no	favorable
RRM1	0.580	1.786	2.73E-01	no	unfavorable
PANX1	-0.583	0.558	2.75E-01	no	favorable
TMEM164	-0.345	0.708	2.76E-01	no	favorable
MYCN	-0.240	0.787	2.77E-01	no	favorable
H3C15	-0.099	0.906	2.80E-01	no	favorable
CIRBP	0.292	1.339	2.80E-01	no	unfavorable
TBX1	-0.221	0.802	2.84E-01	no	favorable
PHKG2	-0.669	0.512	2.86E-01	no	favorable
MAPK14	-0.636	0.530	2.86E-01	no	favorable
HMGB1	0.309	1.361	2.87E-01	no	unfavorable
VCP	0.747	2.110	2.89E-01	no	unfavorable
NR2F2	0.318	1.374	2.89E-01	no	unfavorable
CD38	-0.640	0.527	2.91E-01	no	favorable
PRDX1	0.253	1.288	2.93E-01	no	unfavorable
SATB1	-0.351	0.704	2.94E-01	no	favorable
ELK1	-0.825	0.438	2.95E-01	no	favorable
RICTOR	-0.469	0.626	2.96E-01	no	favorable
ELAVL1	-0.660	0.517	2.96E-01	no	favorable
RB1	-0.654	0.520	2.97E-01	no	favorable
TYRO3	0.355	1.426	3.01E-01	no	unfavorable
HSPA5	0.390	1.477	3.03E-01	no	unfavorable
SND1	0.568	1.765	3.07E-01	no	unfavorable
FNDC3B	-0.316	0.729	3.09E-01	no	favorable
FH	0.558	1.748	3.09E-01	no	unfavorable
IGF2BP3	-0.184	0.832	3.11E-01	no	favorable
HSPB1	0.227	1.255	3.13E-01	no	unfavorable
GPAT4	0.599	1.821	3.16E-01	no	unfavorable
YAP1	0.249	1.283	3.31E-01	no	unfavorable
PTEN	-0.375	0.687	3.32E-01	no	favorable
EGLN2	-0.725	0.485	3.32E-01	no	favorable
OSBPL9	0.572	1.772	3.34E-01	no	unfavorable
CTNNB1	-0.543	0.581	3.34E-01	no	favorable
FUNDC2	0.348	1.416	3.38E-01	no	unfavorable
RNF113A	0.553	1.739	3.38E-01	no	unfavorable
CDC25A	-0.395	0.674	3.41E-01	no	favorable
CHMP5	0.328	1.388	3.43E-01	no	unfavorable

LPCAT3	-0.468	0.626	3.44E-01	no	favorable
DLD	0.394	1.482	3.44E-01	no	unfavorable
CNR1	-0.206	0.814	3.46E-01	no	favorable
CD44	-0.248	0.780	3.48E-01	no	favorable
MMD	-0.261	0.770	3.49E-01	no	favorable
PYCR1	0.349	1.417	3.52E-01	no	unfavorable
NR1D2	-0.417	0.659	3.55E-01	no	favorable
MAPK3	0.585	1.795	3.56E-01	no	unfavorable
NEAT1	0.200	1.221	3.58E-01	no	unfavorable
ATG4D	0.597	1.817	3.68E-01	no	unfavorable
TMBIM4	0.383	1.466	3.70E-01	no	unfavorable
FBLN1	-0.253	0.776	3.70E-01	no	favorable
PTGS2	0.333	1.395	3.72E-01	no	unfavorable
HELLS	-0.281	0.755	3.75E-01	no	favorable
IDH1	0.275	1.317	3.79E-01	no	unfavorable
ETV4	-0.261	0.770	3.81E-01	no	favorable
P2RX7	0.401	1.493	3.82E-01	no	unfavorable
GRN	-0.494	0.610	3.83E-01	no	favorable
COPZ1	0.263	1.301	3.83E-01	no	unfavorable
CASP3	-0.369	0.691	3.84E-01	no	favorable
GLS	0.312	1.366	3.88E-01	no	unfavorable
PARP2	-0.475	0.622	3.88E-01	no	favorable
METTL3	-0.559	0.572	3.90E-01	no	favorable
ATG13	-0.589	0.555	3.91E-01	no	favorable
NOX4	-0.339	0.712	3.96E-01	no	favorable
NR1D1	-0.294	0.745	3.97E-01	no	favorable
IRF7	0.343	1.409	3.97E-01	no	unfavorable
MICU1	0.418	1.519	4.00E-01	no	unfavorable
HOXA11-AS	0.300	1.350	4.01E-01	no	unfavorable
TNFAIP8L2	0.592	1.807	4.01E-01	no	unfavorable
AKR1C1	-0.227	0.797	4.02E-01	no	favorable
APOC1	-0.189	0.828	4.07E-01	no	favorable
CFL1	0.372	1.450	4.08E-01	no	unfavorable
COX7A1	0.179	1.196	4.08E-01	no	unfavorable
MITD1	0.342	1.408	4.10E-01	no	unfavorable
PCDH7	-0.219	0.803	4.12E-01	no	favorable
DELE1	-0.517	0.596	4.13E-01	no	favorable
SEC24B	-0.537	0.585	4.13E-01	no	favorable
HCAR1	-0.227	0.797	4.16E-01	no	favorable
ADIPOR1	0.534	1.705	4.19E-01	no	unfavorable
CARM1	-0.414	0.661	4.23E-01	no	favorable
MEG8	-0.211	0.810	4.26E-01	no	favorable
DHODH	0.411	1.509	4.29E-01	no	unfavorable
CISD1	0.387	1.473	4.30E-01	no	unfavorable

ACSF2	0.215	1.239	4.33E-01	no	unfavorable
MTF1	-0.427	0.653	4.33E-01	no	favorable
FADS2	-0.310	0.734	4.35E-01	no	favorable
PSTK	-0.525	0.591	4.39E-01	no	favorable
EGR1	-0.126	0.881	4.41E-01	no	favorable
SLC16A1-AS1	-0.304	0.738	4.42E-01	no	favorable
CPEB1	0.379	1.460	4.47E-01	no	unfavorable
NFE2L1	0.374	1.453	4.48E-01	no	unfavorable
AXL	0.332	1.394	4.48E-01	no	unfavorable
CDH1	-0.296	0.744	4.49E-01	no	favorable
LAMTOR5	0.202	1.224	4.49E-01	no	unfavorable
SESN2	0.407	1.502	4.50E-01	no	unfavorable
TRIM21	-0.409	0.664	4.52E-01	no	favorable
STEAP3	0.298	1.347	4.54E-01	no	unfavorable
EPHA2	-0.180	0.835	4.54E-01	no	favorable
RIPK3	0.636	1.890	4.55E-01	no	unfavorable
PEX2	0.294	1.342	4.58E-01	no	unfavorable
SLC1A5	0.188	1.207	4.60E-01	no	unfavorable
NFE2L2	0.340	1.405	4.64E-01	no	unfavorable
GCH1	0.597	1.817	4.64E-01	no	unfavorable
ZEB1	-0.255	0.775	4.66E-01	no	favorable
RRM2	-0.217	0.805	4.68E-01	no	favorable
NEDD4L	-0.204	0.815	4.72E-01	no	favorable
ACOT1	-0.355	0.701	4.73E-01	no	favorable
BRD7	-0.279	0.756	4.73E-01	no	favorable
P4HB	0.403	1.496	4.74E-01	no	unfavorable
FUNDC1	-0.269	0.764	4.78E-01	no	favorable
RB1CC1	0.511	1.667	4.80E-01	no	unfavorable
PTPN6	-0.367	0.693	4.81E-01	no	favorable
FAF1	0.367	1.443	4.86E-01	no	unfavorable
DNAJB6	-0.254	0.776	4.88E-01	no	favorable
DPP4	-0.327	0.721	4.90E-01	no	favorable
IRF3	-0.297	0.743	4.96E-01	no	favorable
TERT	-0.279	0.757	4.96E-01	no	favorable
C8orf76	-0.292	0.747	4.99E-01	no	favorable
TUG1	-0.178	0.837	5.05E-01	no	favorable
ATG5	0.377	1.458	5.07E-01	no	unfavorable
PARP1	0.338	1.403	5.08E-01	no	unfavorable
FXN	0.359	1.432	5.09E-01	no	unfavorable
SLC16A1	0.241	1.273	5.16E-01	no	unfavorable
LTBP2	0.168	1.182	5.19E-01	no	unfavorable
TFAM	-0.377	0.686	5.21E-01	no	favorable
IL1B	-0.313	0.731	5.22E-01	no	favorable
HSP90AA1	0.163	1.178	5.23E-01	no	unfavorable

ICA1	-0.274	0.761	5.26E-01	no	favorable
ECH1	0.259	1.295	5.26E-01	no	unfavorable
PARP4	0.299	1.348	5.34E-01	no	unfavorable
HOTAIR	-0.165	0.848	5.35E-01	no	favorable
GLRX5	-0.414	0.661	5.39E-01	no	favorable
TP53	-0.224	0.799	5.41E-01	no	favorable
ARF6	-0.403	0.668	5.48E-01	no	favorable
SOX2	-0.094	0.910	5.48E-01	no	favorable
BACH1	-0.202	0.817	5.49E-01	no	favorable
COQ10B	-0.317	0.728	5.52E-01	no	favorable
CYGB	-0.209	0.812	5.61E-01	no	favorable
QSOX1	0.278	1.320	5.61E-01	no	unfavorable
ARG2	-0.226	0.798	5.67E-01	no	favorable
MFN2	-0.514	0.598	5.69E-01	no	favorable
CRYAB	-0.099	0.906	5.70E-01	no	favorable
NLRP3	-0.541	0.582	5.72E-01	no	favorable
PIR	0.236	1.266	5.75E-01	no	unfavorable
FURIN	0.303	1.353	5.78E-01	no	unfavorable
KRAS	0.246	1.279	5.83E-01	no	unfavorable
PIK3CA	-0.265	0.767	5.87E-01	no	favorable
GABARAPL2	0.202	1.224	5.89E-01	no	unfavorable
ACSL3	0.210	1.234	5.91E-01	no	unfavorable
PRKCB	-0.123	0.884	5.92E-01	no	favorable
PNO1	-0.277	0.758	5.93E-01	no	favorable
ZFP36	0.118	1.125	5.95E-01	no	unfavorable
OIP5-AS1	-0.151	0.860	5.95E-01	no	favorable
MAZ	-0.292	0.747	5.98E-01	no	favorable
PGM5P3-AS1	-0.154	0.857	5.98E-01	no	favorable
HNRNPL	0.412	1.510	6.02E-01	no	unfavorable
PTPMT1	0.207	1.230	6.02E-01	no	unfavorable
KDM5C	-0.329	0.720	6.03E-01	no	favorable
PARP9	-0.188	0.829	6.03E-01	no	favorable
SRSF9	0.301	1.351	6.08E-01	no	unfavorable
PGD	-0.299	0.741	6.14E-01	no	favorable
CISD2	0.158	1.171	6.16E-01	no	unfavorable
MAPKAP1	0.311	1.364	6.18E-01	no	unfavorable
KDM1A	-0.326	0.722	6.19E-01	no	favorable
MUC1	0.229	1.257	6.26E-01	no	unfavorable
TGFB2	0.094	1.099	6.30E-01	no	unfavorable
TRIM46	-0.156	0.855	6.33E-01	no	favorable
BBOX1-AS1	0.174	1.190	6.36E-01	no	unfavorable
TMSB4X	-0.100	0.905	6.37E-01	no	favorable
NKAP	0.314	1.369	6.41E-01	no	unfavorable
SP1	0.315	1.370	6.42E-01	no	unfavorable

BID	0.211	1.235	6.46E-01	no	unfavorable
SCP2	0.178	1.195	6.47E-01	no	unfavorable
LOX	0.148	1.160	6.56E-01	no	unfavorable
ABCC5	-0.194	0.824	6.58E-01	no	favorable
TMEM43	-0.271	0.763	6.59E-01	no	favorable
ATF6	0.294	1.342	6.59E-01	no	unfavorable
BCAT2	-0.187	0.830	6.66E-01	no	favorable
SREBF1	0.148	1.159	6.68E-01	no	unfavorable
G6PD	0.288	1.333	6.71E-01	no	unfavorable
CGAS	0.242	1.274	6.71E-01	no	unfavorable
SLC3A2	0.253	1.287	6.74E-01	no	unfavorable
MYB	-0.141	0.869	6.82E-01	no	favorable
ARPC1A	0.252	1.287	6.83E-01	no	unfavorable
KEAP1	-0.409	0.665	6.90E-01	no	favorable
MDM2	-0.184	0.832	6.90E-01	no	favorable
EPAS1	-0.129	0.879	6.93E-01	no	favorable
SNX4	0.268	1.307	6.94E-01	no	unfavorable
UHRF1	-0.132	0.876	6.94E-01	no	favorable
REST	0.159	1.172	6.98E-01	no	unfavorable
NOD1	0.211	1.234	7.01E-01	no	unfavorable
AKT1S1	0.248	1.281	7.01E-01	no	unfavorable
MAPK1	-0.255	0.775	7.06E-01	no	favorable
PLTP	0.116	1.123	7.06E-01	no	unfavorable
MEG3	0.060	1.062	7.07E-01	no	unfavorable
MIR217	-0.238	0.788	7.16E-01	no	favorable
PRKAA2	-0.203	0.816	7.20E-01	no	favorable
PCBP1	-0.188	0.828	7.23E-01	no	favorable
FZD7	0.078	1.081	7.27E-01	no	unfavorable
ATG3	0.179	1.196	7.36E-01	no	unfavorable
ISCU	0.220	1.246	7.44E-01	no	unfavorable
PDSS2	0.248	1.282	7.45E-01	no	unfavorable
BSG	-0.112	0.894	7.45E-01	no	favorable
MFN1	0.187	1.206	7.50E-01	no	unfavorable
TGFBR1	0.128	1.136	7.51E-01	no	unfavorable
TNFAIP3	0.114	1.121	7.62E-01	no	unfavorable
AEBP1	-0.085	0.918	7.63E-01	no	favorable
IDH2	0.167	1.182	7.73E-01	no	unfavorable
PARP14	-0.126	0.881	7.73E-01	no	favorable
STAT6	0.127	1.135	7.75E-01	no	unfavorable
NR5A2	0.189	1.208	7.80E-01	no	unfavorable
PDGFRA	0.066	1.069	7.81E-01	no	unfavorable
ALOX5	0.116	1.123	7.83E-01	no	unfavorable
NCOA3	0.186	1.204	7.85E-01	no	unfavorable
TRIB2	0.089	1.093	7.90E-01	no	unfavorable

MEF2C	0.110	1.116	7.90E-01	no	unfavorable
EHMT2	-0.173	0.841	7.92E-01	no	favorable
GMFB	0.120	1.127	7.94E-01	no	unfavorable
OTUB1	0.149	1.161	7.99E-01	no	unfavorable
ABCC1	-0.102	0.903	8.06E-01	no	favorable
STAT3	-0.102	0.903	8.08E-01	no	favorable
MARCHF5	-0.151	0.860	8.11E-01	no	favorable
AGAP2-AS1	-0.071	0.932	8.12E-01	no	favorable
ANO1	0.100	1.105	8.18E-01	no	unfavorable
TRPM2	0.151	1.163	8.18E-01	no	unfavorable
TMEM161B-DT	-0.060	0.941	8.22E-01	no	favorable
USP14	0.115	1.122	8.24E-01	no	unfavorable
LRRK2	-0.133	0.875	8.26E-01	no	favorable
TLR4	0.088	1.092	8.33E-01	no	unfavorable
PIEZO1	0.059	1.060	8.35E-01	no	unfavorable
NR4A1	-0.050	0.952	8.38E-01	no	favorable
PGRMC1	0.090	1.094	8.39E-01	no	unfavorable
TF	0.036	1.036	8.45E-01	no	unfavorable
CX3CL1	-0.077	0.926	8.48E-01	no	favorable
EIF4E	-0.073	0.930	8.50E-01	no	favorable
SNCA	-0.048	0.953	8.51E-01	no	favorable
MARCHF6	-0.116	0.891	8.53E-01	no	favorable
ACSL1	0.092	1.096	8.59E-01	no	unfavorable
HMGCL	-0.095	0.909	8.63E-01	no	favorable
SLC11A2	-0.105	0.900	8.69E-01	no	favorable
PRKAA1	-0.097	0.908	8.73E-01	no	favorable
HIF1A	-0.091	0.913	8.81E-01	no	favorable
AGPS	-0.101	0.904	8.81E-01	no	favorable
CPLX2	0.035	1.035	8.92E-01	no	unfavorable
TGFB1	0.035	1.036	8.94E-01	no	unfavorable
LYRM1	-0.054	0.948	8.97E-01	no	favorable
ATG7	0.080	1.083	9.00E-01	no	unfavorable
DDR2	0.036	1.037	9.00E-01	no	unfavorable
PRKCG	-0.048	0.953	9.03E-01	no	favorable
ISCA2	-0.084	0.920	9.05E-01	no	favorable
CYB5R1	0.060	1.062	9.12E-01	no	unfavorable
FADS1	0.049	1.050	9.12E-01	no	unfavorable
ALKBH5	-0.069	0.934	9.13E-01	no	favorable
LINC01606	-0.039	0.962	9.15E-01	no	favorable
RXRG	0.022	1.022	9.17E-01	no	unfavorable
DAZAP1	-0.076	0.927	9.17E-01	no	favorable
AKR1C2	0.020	1.020	9.23E-01	no	unfavorable
HRAS	-0.050	0.952	9.24E-01	no	favorable
GSTZ1	0.041	1.041	9.24E-01	no	unfavorable

NTRK2	-0.021	0.979	9.29E-01	no	favorable
GSTM1	0.015	1.015	9.31E-01	no	unfavorable
SLC40A1	-0.031	0.969	9.31E-01	no	favorable
GPX4	0.061	1.063	9.34E-01	no	unfavorable
CISD3	-0.050	0.951	9.36E-01	no	favorable
CYBB	-0.037	0.964	9.37E-01	no	favorable
TMSB4Y	-0.037	0.964	9.39E-01	no	favorable
ACVR1B	0.053	1.055	9.47E-01	no	unfavorable
CDKN2A	0.020	1.020	9.49E-01	no	unfavorable
SQSTM1	0.035	1.035	9.53E-01	no	unfavorable
FAR1	0.037	1.037	9.56E-01	no	unfavorable
SLC25A28	0.030	1.031	9.59E-01	no	unfavorable
BNC1	-0.017	0.983	9.59E-01	no	favorable
LGMN	0.029	1.029	9.60E-01	no	unfavorable
MAPK9	-0.026	0.974	9.60E-01	no	favorable
EGFR	0.013	1.013	9.63E-01	no	unfavorable
UFL1	-0.024	0.976	9.65E-01	no	favorable
SIRT1	0.026	1.026	9.67E-01	no	unfavorable
PEX6	-0.026	0.975	9.68E-01	no	favorable
CA9	-0.012	0.988	9.72E-01	no	favorable
PPP1R13L	-0.012	0.988	9.72E-01	no	favorable
JUN	-0.008	0.992	9.77E-01	no	favorable
TRIM7	-0.011	0.990	9.85E-01	no	favorable
BAX	0.007	1.007	9.87E-01	no	unfavorable
SRSF1	0.009	1.009	9.88E-01	no	unfavorable
KLF15	-0.005	0.995	9.91E-01	no	favorable
HNRNPD	-0.003	0.997	9.93E-01	no	favorable
WWTR1	-0.002	0.998	9.94E-01	no	favorable
TFRC	0.000	1.000	9.99E-01	no	favorable

Supplemental Table 2. Univariate overall survival Cox analyses performed on expression of ferroptosis related genes in validation cohort.

identifiers	coef.beta	Hazard ratio	p-values	significance	prognosis
PNO1	2.006	7.430	3.60E-08	YES	unfavorable
CCT3	1.831	6.240	7.03E-08	YES	unfavorable
MYC	0.490	1.633	3.62E-07	YES	unfavorable
PYCR1	1.252	3.496	7.63E-07	YES	unfavorable
ALG3	2.097	8.143	9.83E-07	YES	unfavorable
MEN1	2.766	15.897	1.43E-06	YES	unfavorable
DHODH	2.282	9.797	8.20E-06	YES	unfavorable
AHCY	0.895	2.448	1.15E-05	YES	unfavorable
DAZAP1	2.445	11.536	2.85E-05	YES	unfavorable
TFAP2C	0.710	2.035	1.22E-04	YES	unfavorable
TXN	1.110	3.035	1.28E-04	YES	unfavorable
NAT10	1.822	6.183	1.91E-04	YES	unfavorable
PTPMT1	0.686	1.986	1.91E-04	YES	unfavorable
PEX3	-1.811	0.164	2.02E-04	YES	favorable
TBX1	0.426	1.530	3.30E-04	YES	unfavorable
CHAC1	1.005	2.733	3.42E-04	YES	unfavorable
TXNRD1	1.446	4.246	3.88E-04	YES	unfavorable
FADS1	1.300	3.669	4.16E-04	YES	unfavorable
USP11	-1.180	0.307	5.82E-04	YES	favorable
PARP1	1.424	4.153	6.93E-04	YES	unfavorable
HDDC3	1.355	3.876	8.02E-04	YES	unfavorable
SIRT2	-1.715	0.180	9.12E-04	YES	favorable
HSF1	1.185	3.271	9.95E-04	YES	unfavorable
MAP3K11	1.656	5.240	1.05E-	YES	unfavorable

			03		
TCF4	-0.764	0.466	1.20E-03	YES	favorable
VAMP2	-0.754	0.471	1.33E-03	YES	favorable
SOX15	-0.562	0.570	1.37E-03	YES	favorable
ACVR1B	1.302	3.677	1.42E-03	YES	unfavorable
PARP14	0.812	2.253	1.43E-03	YES	unfavorable
COPZ1	1.302	3.677	1.50E-03	YES	unfavorable
MCF2L	-0.518	0.596	1.54E-03	YES	favorable
COQ10A	-0.970	0.379	1.63E-03	YES	favorable
ATF6	1.465	4.329	1.80E-03	YES	unfavorable
MLST8	1.506	4.508	1.83E-03	YES	unfavorable
FAF1	1.385	3.994	2.08E-03	YES	unfavorable
GPX7	0.748	2.113	2.35E-03	YES	unfavorable
ELAVL1	1.521	4.579	2.42E-03	YES	unfavorable
G3BP1	1.016	2.762	2.56E-03	YES	unfavorable
TFAM	1.201	3.323	2.63E-03	YES	unfavorable
MAPK9	-0.992	0.371	2.76E-03	YES	favorable
CAPRIN2	-0.868	0.420	2.79E-03	YES	favorable
SUV39H1	1.601	4.956	3.14E-03	YES	unfavorable
CLOCK	-1.293	0.274	3.67E-03	YES	favorable
ATF4	1.154	3.170	3.68E-03	YES	unfavorable
PRDX4	0.708	2.030	3.91E-03	YES	unfavorable
SLC39A14	0.701	2.016	4.05E-03	YES	unfavorable
CCDC6	0.775	2.170	4.63E-03	YES	unfavorable
PML	1.410	4.094	4.73E-03	YES	unfavorable
CEMIP	-0.506	0.603	4.75E-	YES	favorable

			03		
PSEN2	1.150	3.160	4.80E-03	YES	unfavorable
CARS1	0.826	2.285	5.00E-03	YES	unfavorable
BCAT2	0.541	1.718	5.90E-03	YES	unfavorable
USF2	-1.247	0.287	6.00E-03	YES	favorable
STC1	0.665	1.944	6.01E-03	YES	unfavorable
H3C10	0.546	1.727	6.78E-03	YES	unfavorable
ICA1	0.683	1.980	7.06E-03	YES	unfavorable
TMSB4X	-0.567	0.567	7.28E-03	YES	favorable
CBS	0.559	1.749	7.42E-03	YES	unfavorable
ATG16L1	-1.292	0.275	7.93E-03	YES	favorable
PARP9	0.488	1.629	8.20E-03	YES	unfavorable
SQOR	1.092	2.981	8.30E-03	YES	unfavorable
SDHD	1.227	3.410	8.62E-03	YES	unfavorable
CDO1	-0.456	0.634	8.91E-03	YES	favorable
TGFB1	0.431	1.539	9.48E-03	YES	unfavorable
TSC1	-0.546	0.579	1.02E-02	YES	favorable
TP53	0.825	2.281	1.04E-02	YES	unfavorable
HPX	-0.399	0.671	1.11E-02	YES	favorable
ATG7	1.292	3.640	1.12E-02	YES	unfavorable
FTL	-0.883	0.414	1.12E-02	YES	favorable
FAM98A	0.942	2.566	1.22E-02	YES	unfavorable
AQP4	-0.252	0.777	1.23E-02	YES	favorable
AKT1S1	0.732	2.079	1.25E-02	YES	unfavorable
RBM24	-0.258	0.772	1.28E-02	YES	favorable
AURKA	0.966	2.627	1.29E-	YES	unfavorable

			02		
PARP6	-0.780	0.458	1.33E-02	YES	favorable
MAZ	1.219	3.384	1.34E-02	YES	unfavorable
NR1D2	-0.765	0.465	1.36E-02	YES	favorable
SDHC	1.081	2.948	1.37E-02	YES	unfavorable
PLTP	0.387	1.473	1.37E-02	YES	unfavorable
FXN	0.646	1.909	1.38E-02	YES	unfavorable
PPARD	-1.148	0.317	1.38E-02	YES	favorable
VCP	1.566	4.788	1.43E-02	YES	unfavorable
FXR1	1.217	3.378	1.46E-02	YES	unfavorable
MTDH	0.726	2.066	1.49E-02	YES	unfavorable
SRSF1	1.055	2.873	1.53E-02	YES	unfavorable
PCDH7	-0.547	0.579	1.59E-02	YES	favorable
PEX2	0.702	2.017	1.63E-02	YES	unfavorable
PEX6	-0.940	0.390	1.65E-02	YES	favorable
BID	0.790	2.203	1.78E-02	YES	unfavorable
PANX1	0.584	1.793	1.81E-02	YES	unfavorable
TNFAIP1	-0.905	0.404	1.91E-02	YES	favorable
BEX1	-0.629	0.533	1.93E-02	YES	favorable
OTUB1	1.389	4.012	1.94E-02	YES	unfavorable
CUL9	-0.536	0.585	2.10E-02	YES	favorable
PGD	1.163	3.199	2.13E-02	YES	unfavorable
KIF20A	0.636	1.889	2.13E-02	YES	unfavorable
PCDHB14	-0.491	0.612	2.23E-02	YES	favorable
DNAJB6	-0.698	0.497	2.39E-02	YES	favorable
BMAL1	-0.509	0.601	2.43E-	YES	favorable

			02		
RXRG	-0.246	0.782	2.43E-02	YES	favorable
PTEN	-0.638	0.528	2.49E-02	YES	favorable
SNX5	1.034	2.812	2.74E-02	YES	unfavorable
MAP1LC3A	-0.523	0.593	2.77E-02	YES	favorable
SLC40A1	0.405	1.500	2.79E-02	YES	unfavorable
ADAM23	-0.516	0.597	2.86E-02	YES	favorable
MITF	0.516	1.675	2.87E-02	YES	unfavorable
TRPV1	-0.599	0.549	2.90E-02	YES	favorable
ABCC1	0.787	2.197	2.96E-02	YES	unfavorable
PROM2	-0.624	0.536	2.96E-02	YES	favorable
IL6	0.522	1.685	3.10E-02	YES	unfavorable
TFR2	-0.492	0.611	3.17E-02	YES	favorable
HSPA5	0.808	2.242	3.30E-02	YES	unfavorable
SLC1A5	0.497	1.644	3.50E-02	YES	unfavorable
PPP1R13L	0.436	1.547	3.62E-02	YES	unfavorable
MTOR	-0.890	0.411	3.63E-02	YES	favorable
CAV1	0.558	1.747	3.66E-02	YES	unfavorable
NTRK2	-0.295	0.745	3.71E-02	YES	favorable
FURIN	0.825	2.281	3.93E-02	YES	unfavorable
TIMM9	0.685	1.983	3.98E-02	YES	unfavorable
CRYAB	-0.237	0.789	4.01E-02	YES	favorable
PARP2	-0.885	0.413	4.02E-02	YES	favorable
PHGDH	0.465	1.592	4.04E-02	YES	unfavorable
SMG9	-0.897	0.408	4.19E-02	YES	favorable
P4HB	1.042	2.834	4.20E-	YES	unfavorable

			02		
UBIAD1	1.046	2.845	4.28E-02	YES	unfavorable
PPARA	-0.718	0.488	4.33E-02	YES	favorable
CISD3	0.587	1.798	4.64E-02	YES	unfavorable
BSG	0.450	1.568	4.66E-02	YES	unfavorable
NT5DC2	0.491	1.635	4.68E-02	YES	unfavorable
GRN	0.806	2.238	4.69E-02	YES	unfavorable
CDC25A	0.545	1.724	4.71E-02	YES	unfavorable
PRR5	-0.379	0.684	4.88E-02	YES	favorable
SESN2	0.774	2.169	4.88E-02	YES	unfavorable
PRDX1	0.294	1.341	5.15E-02	no	unfavorable
SLC25A37	0.510	1.665	5.41E-02	no	unfavorable
SIRT6	-1.100	0.333	5.61E-02	no	favorable
KDM4A	0.877	2.403	5.65E-02	no	unfavorable
ADAMTS13	-0.378	0.685	5.71E-02	no	favorable
CIRBP	-0.428	0.652	5.77E-02	no	favorable
SIAH2	0.803	2.232	5.80E-02	no	unfavorable
LRP8	-0.407	0.666	6.04E-02	no	favorable
ACO1	1.074	2.927	6.07E-02	no	unfavorable
FH	0.857	2.355	6.10E-02	no	unfavorable
MTCH1	-1.231	0.292	6.51E-02	no	favorable
PTPN6	0.689	1.991	6.52E-02	no	unfavorable
DDIT4	0.461	1.585	6.52E-02	no	unfavorable
SCD	0.439	1.551	6.53E-02	no	unfavorable
SLC7A11	0.330	1.391	6.63E-02	no	unfavorable
RNF113A	0.828	2.289	6.70E-	no	unfavorable

			02		
ANO6	0.790	2.204	6.79E-02	no	unfavorable
RELA	0.879	2.410	6.80E-02	no	unfavorable
YTHDC2	-0.675	0.509	6.90E-02	no	favorable
POR	-0.657	0.519	6.94E-02	no	favorable
CD82	0.287	1.332	7.08E-02	no	unfavorable
AGPS	0.768	2.156	7.17E-02	no	unfavorable
CAMKK2	0.483	1.622	7.22E-02	no	unfavorable
COMMD10	0.657	1.928	7.29E-02	no	unfavorable
RICTOR	-0.724	0.485	7.33E-02	no	favorable
GABARAPL1	-0.403	0.668	7.39E-02	no	favorable
KDM3B	-1.105	0.331	7.46E-02	no	favorable
FBXW7	-0.541	0.582	7.53E-02	no	favorable
AXL	0.607	1.835	7.61E-02	no	unfavorable
KAT5	0.809	2.245	7.63E-02	no	unfavorable
ATP5MC3	0.991	2.693	7.68E-02	no	unfavorable
PDIA2	0.169	1.184	7.72E-02	no	unfavorable
PCBP1	1.232	3.429	7.75E-02	no	unfavorable
MUC1	0.294	1.342	8.01E-02	no	unfavorable
MAPKAP1	0.911	2.486	8.33E-02	no	unfavorable
GDF15	0.735	2.086	8.39E-02	no	unfavorable
SND1	0.583	1.792	8.43E-02	no	unfavorable
RRM2	0.432	1.541	8.76E-02	no	unfavorable
SSBP1	0.599	1.820	8.89E-02	no	unfavorable
RPTOR	0.953	2.595	8.92E-02	no	unfavorable
STING1	0.561	1.753	8.98E-	no	unfavorable

			02		
CA9	0.496	1.643	9.15E-02	no	unfavorable
CTNNB1	-0.602	0.548	9.25E-02	no	favorable
ACADSB	-0.584	0.558	9.28E-02	no	favorable
FTO	-0.568	0.567	9.30E-02	no	favorable
RND1	-0.236	0.790	9.34E-02	no	favorable
KLF15	0.401	1.493	9.85E-02	no	unfavorable
BRCA1	0.546	1.726	9.89E-02	no	unfavorable
ALOX12	-0.534	0.586	1.01E-01	no	favorable
CPEB1	0.371	1.450	1.01E-01	no	unfavorable
CISD1	0.536	1.709	1.01E-01	no	unfavorable
AKR1C2	-0.253	0.776	1.05E-01	no	favorable
PIR	0.474	1.607	1.08E-01	no	unfavorable
POU2F2	-0.281	0.755	1.08E-01	no	favorable
ATF2	-0.803	0.448	1.08E-01	no	favorable
GLRX5	0.562	1.755	1.08E-01	no	unfavorable
MARCHF6	-0.705	0.494	1.09E-01	no	favorable
CNR1	-0.192	0.825	1.11E-01	no	favorable
CS	0.863	2.371	1.13E-01	no	unfavorable
SLC16A1	0.374	1.454	1.14E-01	no	unfavorable
HPCAL1	-0.316	0.729	1.15E-01	no	favorable
SLC38A1	-0.268	0.765	1.15E-01	no	favorable
AKR1C1	-0.327	0.721	1.15E-01	no	favorable
PARP11	0.590	1.804	1.16E-01	no	unfavorable
PARP4	0.624	1.866	1.17E-01	no	unfavorable
ARF6	0.738	2.091	1.17E-	no	unfavorable

			01		
ULK1	-0.530	0.589	1.18E-01	no	favorable
RAPGEF3	-0.342	0.710	1.18E-01	no	favorable
GSTZ1	0.514	1.672	1.18E-01	no	unfavorable
DKK1	0.567	1.762	1.21E-01	no	unfavorable
PGRMC1	-0.569	0.566	1.21E-01	no	favorable
CISD2	0.656	1.927	1.24E-01	no	unfavorable
PEX10	0.731	2.077	1.24E-01	no	unfavorable
TRIM21	0.445	1.561	1.24E-01	no	unfavorable
BECN1	-0.684	0.504	1.25E-01	no	favorable
FTH1	-0.423	0.655	1.26E-01	no	favorable
OSBPL9	0.697	2.007	1.26E-01	no	unfavorable
CD44	0.383	1.467	1.30E-01	no	unfavorable
PDSS2	0.983	2.672	1.31E-01	no	unfavorable
SP1	0.769	2.158	1.33E-01	no	unfavorable
CTSB	0.535	1.707	1.36E-01	no	unfavorable
EZH2	-0.344	0.709	1.37E-01	no	favorable
TRIB2	0.248	1.282	1.37E-01	no	unfavorable
CARM1	0.796	2.216	1.37E-01	no	unfavorable
SREBF1	0.362	1.436	1.41E-01	no	unfavorable
ATG4D	0.555	1.742	1.41E-01	no	unfavorable
HUWE1	-0.602	0.547	1.45E-01	no	favorable
SAT1	-0.652	0.521	1.48E-01	no	favorable
NFE2L1	0.392	1.480	1.49E-01	no	unfavorable
KDM6B	-0.331	0.718	1.49E-01	no	favorable
YAP1	0.263	1.301	1.50E-	no	unfavorable

			01		
FNDC3B	0.462	1.588	1.50E-01	no	unfavorable
MIB2	-0.389	0.678	1.54E-01	no	favorable
RRM1	0.532	1.702	1.55E-01	no	unfavorable
ELOVL5	0.695	2.003	1.56E-01	no	unfavorable
ISCU	-0.904	0.405	1.56E-01	no	favorable
HELLS	0.494	1.639	1.58E-01	no	unfavorable
CREB5	0.339	1.403	1.58E-01	no	unfavorable
HSPA8	0.443	1.557	1.60E-01	no	unfavorable
AEBP2	0.719	2.052	1.60E-01	no	unfavorable
ACSL4	-0.443	0.642	1.61E-01	no	favorable
HRAS	0.425	1.530	1.64E-01	no	unfavorable
ENPP2	0.128	1.136	1.67E-01	no	unfavorable
SATB1	-0.288	0.750	1.68E-01	no	favorable
PLIN2	0.477	1.612	1.69E-01	no	unfavorable
NR1D1	-0.336	0.715	1.69E-01	no	favorable
G6PD	0.742	2.100	1.72E-01	no	unfavorable
AGPAT3	0.492	1.635	1.72E-01	no	unfavorable
HIF1A	-0.526	0.591	1.73E-01	no	favorable
FSCN1	-0.273	0.761	1.74E-01	no	favorable
NOTCH3	0.248	1.281	1.74E-01	no	unfavorable
EGR1	-0.174	0.840	1.80E-01	no	favorable
NUP62	0.604	1.830	1.81E-01	no	unfavorable
SMAD7	-0.348	0.706	1.82E-01	no	favorable
HNRNPL	0.866	2.377	1.83E-01	no	unfavorable
ATF3	0.302	1.353	1.84E-	no	unfavorable

			01		
DECR1	0.356	1.428	1.85E-01	no	unfavorable
HERPUD1	0.531	1.700	1.89E-01	no	unfavorable
MICU1	-0.454	0.635	1.89E-01	no	favorable
NEDD4	-0.298	0.742	1.97E-01	no	favorable
ISCA2	0.443	1.558	1.98E-01	no	unfavorable
PEDS1	0.472	1.603	1.99E-01	no	unfavorable
BCAT1	0.287	1.333	2.00E-01	no	unfavorable
STEAP3	0.283	1.327	2.01E-01	no	unfavorable
VDAC2	0.723	2.061	2.01E-01	no	unfavorable
TAFAZZIN	-0.428	0.652	2.01E-01	no	favorable
GRSF1	0.684	1.981	2.10E-01	no	unfavorable
MXRA8	-0.164	0.849	2.15E-01	no	favorable
RBMS1	0.300	1.350	2.15E-01	no	unfavorable
MAPK14	-0.543	0.581	2.18E-01	no	favorable
SLC3A2	0.453	1.573	2.19E-01	no	unfavorable
DUOX1	-0.241	0.786	2.23E-01	no	favorable
AQP11	0.567	1.762	2.27E-01	no	unfavorable
ALKBH5	-0.391	0.677	2.29E-01	no	favorable
PRKCA	-0.418	0.658	2.34E-01	no	favorable
CAVIN1	0.222	1.249	2.34E-01	no	unfavorable
LGMN	0.534	1.706	2.37E-01	no	unfavorable
SMPD1	-0.450	0.638	2.38E-01	no	favorable
PHF21A	-0.520	0.594	2.42E-01	no	favorable
NQO1	0.322	1.380	2.42E-01	no	unfavorable
MFN2	-0.602	0.548	2.44E-	no	favorable

			01		
RARRES2	0.174	1.190	2.44E-01	no	unfavorable
METTL16	-0.347	0.707	2.45E-01	no	favorable
GSTM1	0.128	1.136	2.45E-01	no	unfavorable
EIF2AK2	0.726	2.067	2.46E-01	no	unfavorable
HMOX1	0.307	1.359	2.48E-01	no	unfavorable
TF	-0.180	0.835	2.54E-01	no	favorable
GSK3B	-0.551	0.577	2.54E-01	no	favorable
USP22	-0.276	0.759	2.54E-01	no	favorable
EGLN2	-0.438	0.645	2.62E-01	no	favorable
SDHB	0.679	1.973	2.67E-01	no	unfavorable
PRDX6	0.255	1.291	2.69E-01	no	unfavorable
STAT6	0.276	1.318	2.71E-01	no	unfavorable
LTBP2	0.220	1.246	2.73E-01	no	unfavorable
PLA2G6	-0.323	0.724	2.73E-01	no	favorable
UHRF1	0.259	1.296	2.80E-01	no	unfavorable
TRIM59	0.337	1.400	2.82E-01	no	unfavorable
HILPDA	0.432	1.540	2.82E-01	no	unfavorable
MAPK3	0.442	1.556	2.83E-01	no	unfavorable
APOC1	-0.218	0.804	2.85E-01	no	favorable
NSUN5	0.520	1.683	2.85E-01	no	unfavorable
SNCA	-0.174	0.841	2.86E-01	no	favorable
IGF2BP3	0.141	1.152	2.86E-01	no	unfavorable
KDM5A	0.391	1.478	2.86E-01	no	unfavorable
TBK1	-0.700	0.497	2.87E-01	no	favorable
ATG13	0.445	1.561	2.91E-	no	unfavorable

			01		
ABHD12	0.386	1.471	2.95E-01	no	unfavorable
TGFB2	0.186	1.204	2.97E-01	no	unfavorable
NCOA3	0.563	1.756	3.01E-01	no	unfavorable
ANGPTL4	0.236	1.267	3.02E-01	no	unfavorable
STAT3	-0.251	0.778	3.05E-01	no	favorable
FBLN1	-0.205	0.815	3.09E-01	no	favorable
SELENOP	0.174	1.190	3.10E-01	no	unfavorable
FBXO31	-0.220	0.802	3.12E-01	no	favorable
MFN1	-0.338	0.713	3.14E-01	no	favorable
C8orf76	0.354	1.424	3.17E-01	no	unfavorable
IRF7	0.246	1.279	3.20E-01	no	unfavorable
TGFBR1	0.185	1.203	3.21E-01	no	unfavorable
HSPB1	0.206	1.229	3.25E-01	no	unfavorable
APP	-0.191	0.826	3.26E-01	no	favorable
RPL8	0.310	1.363	3.28E-01	no	unfavorable
TRIM7	-0.265	0.767	3.30E-01	no	favorable
NEDD4L	0.274	1.315	3.30E-01	no	unfavorable
FOXO3	-0.457	0.633	3.31E-01	no	favorable
METTL3	-0.373	0.688	3.32E-01	no	favorable
PDGFRA	0.206	1.229	3.32E-01	no	unfavorable
SEC24B	-0.534	0.586	3.35E-01	no	favorable
NOD1	0.413	1.511	3.37E-01	no	unfavorable
CFL1	-0.373	0.689	3.40E-01	no	favorable
TFRC	0.273	1.314	3.40E-01	no	unfavorable
GCLC	0.230	1.259	3.46E-	no	unfavorable

			01		
ULK2	-0.238	0.788	3.50E-01	no	favorable
ATG5	0.502	1.652	3.52E-01	no	unfavorable
ACSL3	-0.332	0.718	3.52E-01	no	favorable
PTGS2	0.258	1.295	3.61E-01	no	unfavorable
IDH2	0.316	1.372	3.64E-01	no	unfavorable
TRIM46	-0.159	0.853	3.65E-01	no	favorable
BAX	0.308	1.361	3.66E-01	no	unfavorable
CPLX2	-0.128	0.880	3.68E-01	no	favorable
ACOT1	-0.266	0.767	3.68E-01	no	favorable
CYB5R1	0.343	1.410	3.69E-01	no	unfavorable
ETHE1	-0.353	0.703	3.72E-01	no	favorable
KMT2B	-0.301	0.740	3.76E-01	no	favorable
FANCD2	0.243	1.275	3.77E-01	no	unfavorable
ENO3	0.247	1.280	3.81E-01	no	unfavorable
FOXO4	0.290	1.336	3.81E-01	no	unfavorable
MMD	0.196	1.216	3.86E-01	no	unfavorable
SRC	-0.226	0.798	3.88E-01	no	favorable
MPC1	-0.360	0.698	3.93E-01	no	favorable
AQP3	-0.231	0.793	3.99E-01	no	favorable
CDH1	0.231	1.259	4.00E-01	no	unfavorable
EGFR	-0.182	0.833	4.00E-01	no	favorable
GABARAPL2	-0.254	0.776	4.03E-01	no	favorable
GMFB	0.309	1.363	4.04E-01	no	unfavorable
GPX4	-0.412	0.662	4.04E-01	no	favorable
REST	0.247	1.280	4.10E-	no	unfavorable

			01		
LONP1	0.441	1.555	4.13E-01	no	unfavorable
SLC25A28	-0.299	0.742	4.14E-01	no	favorable
MDM2	-0.217	0.805	4.18E-01	no	favorable
CDKN1A	0.153	1.165	4.22E-01	no	unfavorable
GPAT4	0.299	1.349	4.25E-01	no	unfavorable
GGT1	0.365	1.441	4.25E-01	no	unfavorable
NR4A1	0.161	1.175	4.27E-01	no	unfavorable
MIB1	-0.298	0.742	4.29E-01	no	favorable
CYBB	0.314	1.369	4.30E-01	no	unfavorable
FUNDC1	-0.270	0.763	4.30E-01	no	favorable
KDM1A	0.341	1.407	4.36E-01	no	unfavorable
LPIN1	-0.155	0.857	4.37E-01	no	favorable
CHMP6	-0.319	0.727	4.38E-01	no	favorable
CDC27	0.314	1.369	4.40E-01	no	unfavorable
PSTK	-0.365	0.694	4.42E-01	no	favorable
IREB2	0.374	1.453	4.48E-01	no	unfavorable
EPHA2	0.108	1.114	4.48E-01	no	unfavorable
PIEZO1	0.136	1.145	4.50E-01	no	unfavorable
RB1CC1	-0.293	0.746	4.52E-01	no	favorable
SLC25A11	-0.250	0.779	4.55E-01	no	favorable
BMP4	-0.111	0.895	4.57E-01	no	favorable
TNFAIP3	0.259	1.296	4.58E-01	no	unfavorable
NR2F2	0.110	1.116	4.58E-01	no	unfavorable
GJA1	0.111	1.117	4.62E-01	no	unfavorable
ELK1	0.367	1.444	4.64E-	no	unfavorable

			01		
PDK4	0.142	1.153	4.65E-01	no	unfavorable
LYRM1	0.265	1.303	4.66E-01	no	unfavorable
KEAP1	0.291	1.337	4.67E-01	no	unfavorable
PSEN1	0.338	1.402	4.72E-01	no	unfavorable
CYGB	0.158	1.171	4.73E-01	no	unfavorable
UFL1	0.253	1.288	4.74E-01	no	unfavorable
USP14	0.413	1.511	4.74E-01	no	unfavorable
CHMP1A	0.272	1.312	4.74E-01	no	unfavorable
SRSF9	0.349	1.417	4.78E-01	no	unfavorable
TMEM43	-0.319	0.727	4.81E-01	no	favorable
ME1	0.283	1.327	4.82E-01	no	unfavorable
HMGCL	0.303	1.354	4.84E-01	no	unfavorable
LIFR	0.205	1.228	4.86E-01	no	unfavorable
TMBIM4	-0.339	0.712	4.89E-01	no	favorable
SOCS1	-0.177	0.838	4.91E-01	no	favorable
MEF2C	-0.172	0.842	4.98E-01	no	favorable
QSOX1	-0.282	0.754	5.00E-01	no	favorable
NUAK2	0.209	1.233	5.00E-01	no	unfavorable
DDR2	0.165	1.180	5.01E-01	no	unfavorable
NPC1	-0.386	0.680	5.03E-01	no	favorable
DLD	0.243	1.275	5.05E-01	no	unfavorable
AEBP1	-0.145	0.865	5.06E-01	no	favorable
TRPM7	0.284	1.328	5.09E-01	no	unfavorable
SIRT3	-0.228	0.796	5.15E-01	no	favorable
KMT2D	-0.170	0.844	5.16E-	no	favorable

			01		
CERK	-0.162	0.851	5.18E-01	no	favorable
CREB3	-0.325	0.722	5.22E-01	no	favorable
CP	-0.112	0.894	5.36E-01	no	favorable
MDM4	-0.194	0.824	5.37E-01	no	favorable
NDRG1	0.122	1.130	5.44E-01	no	unfavorable
CX3CL1	0.120	1.128	5.46E-01	no	unfavorable
PARP3	0.244	1.276	5.47E-01	no	unfavorable
CD38	0.258	1.294	5.54E-01	no	unfavorable
KLHDC3	-0.357	0.700	5.56E-01	no	favorable
PTPN18	-0.157	0.855	5.58E-01	no	favorable
PER1	0.116	1.123	5.59E-01	no	unfavorable
LOX	0.106	1.112	5.63E-01	no	unfavorable
CDKN2A	-0.120	0.887	5.66E-01	no	favorable
SLC11A2	0.310	1.363	5.66E-01	no	unfavorable
HDLBP	0.208	1.232	5.68E-01	no	unfavorable
AQP5	-0.057	0.944	5.71E-01	no	favorable
S100A8	-0.081	0.922	5.73E-01	no	favorable
PCSK9	0.174	1.190	5.74E-01	no	unfavorable
NRAS	0.291	1.338	5.74E-01	no	unfavorable
PEX12	-0.185	0.831	5.79E-01	no	favorable
FADS2	0.148	1.160	5.80E-01	no	unfavorable
PIK3CA	0.204	1.226	5.83E-01	no	unfavorable
SCP2	-0.182	0.834	5.88E-01	no	favorable
SHARPIN	0.196	1.217	5.89E-01	no	unfavorable
MITD1	-0.271	0.763	5.90E-	no	favorable

			01		
OMA1	0.211	1.235	5.91E-01	no	unfavorable
ZSCAN25	0.244	1.277	5.95E-01	no	unfavorable
NF2	0.239	1.270	6.01E-01	no	unfavorable
ACSL1	-0.151	0.860	6.04E-01	no	favorable
BRPF1	0.250	1.284	6.12E-01	no	unfavorable
PAQR3	-0.163	0.849	6.13E-01	no	favorable
HMGB1	-0.178	0.837	6.24E-01	no	favorable
PCBP2	-0.253	0.777	6.26E-01	no	favorable
MLLT1	-0.239	0.788	6.28E-01	no	favorable
ETV4	-0.127	0.881	6.29E-01	no	favorable
RB1	0.201	1.223	6.43E-01	no	unfavorable
NCOA4	0.205	1.228	6.48E-01	no	unfavorable
INTS2	0.211	1.235	6.50E-01	no	unfavorable
WIPI2	-0.231	0.794	6.55E-01	no	favorable
KDM5C	-0.186	0.830	6.55E-01	no	favorable
AIFM2	-0.216	0.806	6.61E-01	no	favorable
H3C2	0.103	1.108	6.64E-01	no	unfavorable
SNAI2	-0.082	0.921	6.70E-01	no	favorable
MTF1	0.238	1.269	6.74E-01	no	unfavorable
CASP3	0.126	1.134	6.80E-01	no	unfavorable
MCU	0.200	1.222	6.81E-01	no	unfavorable
STK11	0.237	1.268	6.88E-01	no	unfavorable
EIF4E	0.127	1.135	6.89E-01	no	unfavorable
PHKG2	-0.191	0.826	6.94E-01	no	favorable
ZEB1	-0.068	0.934	6.98E-	no	favorable

			01		
GOT1	0.137	1.147	6.98E-01	no	unfavorable
NKAP	0.185	1.204	7.01E-01	no	unfavorable
ZFP36	-0.063	0.939	7.02E-01	no	favorable
IRF3	-0.122	0.885	7.05E-01	no	favorable
WWTR1	0.096	1.101	7.10E-01	no	unfavorable
TULP1	0.022	1.022	7.11E-01	no	unfavorable
SREBF2	0.105	1.111	7.13E-01	no	unfavorable
KAT6B	-0.119	0.888	7.15E-01	no	favorable
ENO1	0.124	1.133	7.18E-01	no	unfavorable
LIG3	0.110	1.117	7.21E-01	no	unfavorable
TOR2A	0.142	1.152	7.21E-01	no	unfavorable
WIPI1	0.119	1.127	7.23E-01	no	unfavorable
SDHA	0.164	1.179	7.24E-01	no	unfavorable
LIN28A	0.128	1.136	7.26E-01	no	unfavorable
ATM	0.134	1.143	7.30E-01	no	unfavorable
CDCA3	-0.105	0.901	7.34E-01	no	favorable
HLF	0.068	1.070	7.35E-01	no	unfavorable
APOE	-0.055	0.946	7.36E-01	no	favorable
BAP1	-0.183	0.833	7.38E-01	no	favorable
CD74	0.067	1.069	7.40E-01	no	unfavorable
PARP8	0.087	1.091	7.43E-01	no	unfavorable
CREB1	-0.123	0.884	7.47E-01	no	favorable
MYB	0.112	1.118	7.52E-01	no	unfavorable
SUFU	0.126	1.134	7.55E-01	no	unfavorable
NTN1	0.040	1.041	7.55E-	no	unfavorable

			01		
ASAP2	-0.087	0.917	7.56E-01	no	favorable
PIM1	0.097	1.101	7.56E-01	no	unfavorable
ADIPOR1	0.158	1.171	7.57E-01	no	unfavorable
COQ10B	-0.153	0.858	7.59E-01	no	favorable
MAP3K14	-0.063	0.939	7.59E-01	no	favorable
GLS	0.103	1.108	7.59E-01	no	unfavorable
COX7A1	-0.037	0.964	7.62E-01	no	favorable
MARCHF5	0.117	1.124	7.64E-01	no	unfavorable
ERN1	0.099	1.104	7.66E-01	no	unfavorable
TRIM26	-0.059	0.943	7.71E-01	no	favorable
BRD3	-0.105	0.900	7.76E-01	no	favorable
SOCS2	-0.087	0.916	7.76E-01	no	favorable
FUNDC2	0.114	1.120	7.76E-01	no	unfavorable
JUN	0.062	1.064	7.79E-01	no	unfavorable
TIMP1	-0.047	0.954	7.84E-01	no	favorable
BRD4	-0.101	0.904	7.91E-01	no	favorable
ATG3	0.151	1.163	7.91E-01	no	unfavorable
CHMP5	0.132	1.142	7.92E-01	no	unfavorable
MYCN	-0.033	0.967	7.93E-01	no	favorable
PARP12	0.050	1.051	7.94E-01	no	unfavorable
PRDX2	-0.114	0.892	7.98E-01	no	favorable
FNDC5	0.044	1.045	7.99E-01	no	unfavorable
SIRT1	-0.108	0.898	8.02E-01	no	favorable
SPHK1	0.051	1.052	8.04E-01	no	unfavorable
NUPR1	-0.064	0.938	8.04E-	no	favorable

			01		
IDH1	-0.068	0.935	8.04E-01	no	favorable
DELE1	-0.074	0.929	8.05E-01	no	favorable
HNRNPD	0.115	1.122	8.06E-01	no	unfavorable
SNX4	0.121	1.129	8.10E-01	no	unfavorable
ALDH3A2	0.072	1.075	8.12E-01	no	unfavorable
USP7	-0.111	0.895	8.14E-01	no	favorable
FGFR4	0.095	1.100	8.16E-01	no	unfavorable
FZD7	0.033	1.034	8.18E-01	no	unfavorable
C19orf12	-0.117	0.890	8.18E-01	no	favorable
MTHFR	-0.086	0.917	8.18E-01	no	favorable
ACSF2	0.061	1.063	8.21E-01	no	unfavorable
SOX2	0.033	1.034	8.26E-01	no	unfavorable
USP35	0.049	1.051	8.27E-01	no	unfavorable
EHMT2	0.052	1.053	8.27E-01	no	unfavorable
LAMTOR5	0.098	1.102	8.29E-01	no	unfavorable
DCAF7	-0.077	0.926	8.30E-01	no	favorable
CHP1	0.099	1.104	8.31E-01	no	unfavorable
PEBP1	-0.069	0.933	8.32E-01	no	favorable
TYRO3	0.067	1.070	8.33E-01	no	unfavorable
HMGCR	0.073	1.076	8.36E-01	no	unfavorable
PARP10	0.065	1.067	8.41E-01	no	unfavorable
SIRT7	0.085	1.089	8.48E-01	no	unfavorable
LPCAT3	0.051	1.053	8.51E-01	no	unfavorable
BACH1	-0.067	0.935	8.53E-01	no	favorable
HSP90AA1	0.050	1.051	8.60E-	no	unfavorable

			01		
MT1G	-0.037	0.963	8.68E-01	no	favorable
KRAS	0.066	1.068	8.68E-01	no	unfavorable
NFS1	-0.076	0.927	8.71E-01	no	favorable
BRD2	-0.041	0.960	8.73E-01	no	favorable
FAR1	0.069	1.072	8.77E-01	no	unfavorable
JUND	0.044	1.045	8.82E-01	no	unfavorable
EMC2	0.036	1.037	8.84E-01	no	unfavorable
PARK7	0.074	1.077	8.92E-01	no	unfavorable
PARP16	0.079	1.082	8.94E-01	no	unfavorable
SQSTM1	0.043	1.044	9.03E-01	no	unfavorable
ABCC5	-0.031	0.970	9.06E-01	no	favorable
BRD7	-0.057	0.944	9.10E-01	no	favorable
TIGAR	-0.014	0.986	9.10E-01	no	favorable
KLF2	-0.028	0.973	9.15E-01	no	favorable
MAPK8	-0.029	0.971	9.15E-01	no	favorable
AKR1C3	0.029	1.030	9.17E-01	no	unfavorable
METTL14	0.031	1.032	9.18E-01	no	unfavorable
YY1AP1	0.060	1.061	9.18E-01	no	unfavorable
PRKAA1	0.035	1.035	9.29E-01	no	unfavorable
SPATA2	-0.057	0.945	9.31E-01	no	favorable
MGST1	0.019	1.019	9.36E-01	no	unfavorable
ECH1	-0.043	0.958	9.36E-01	no	favorable
SENP1	0.040	1.040	9.37E-01	no	unfavorable
ARG2	-0.018	0.982	9.46E-01	no	favorable
JUP	-0.014	0.986	9.49E-	no	favorable

			01		
SLC39A7	-0.026	0.975	9.54E-01	no	favorable
PEX14	0.025	1.025	9.57E-01	no	unfavorable
ARMC5	-0.019	0.981	9.60E-01	no	favorable
PER2	-0.016	0.985	9.60E-01	no	favorable
EPAS1	0.008	1.008	9.66E-01	no	unfavorable
NFE2L2	-0.017	0.983	9.67E-01	no	favorable
ARPC1A	0.014	1.014	9.70E-01	no	unfavorable
KRT18	0.010	1.010	9.74E-01	no	unfavorable
TMEM164	-0.006	0.994	9.75E-01	no	favorable
EMP1	-0.004	0.996	9.76E-01	no	favorable
TFAP2A	-0.006	0.994	9.77E-01	no	favorable
LAMP2	-0.010	0.990	9.79E-01	no	favorable
MAPK1	-0.008	0.992	9.81E-01	no	favorable
GALNT14	0.001	1.001	9.89E-01	no	unfavorable

Disclaimer/Publisher's Note: The statements, opinions and data contained in all publications are solely those of the individual author(s) and contributor(s) and not of MDPI and/or the editor(s). MDPI and/or the editor(s) disclaim responsibility for any injury to people or property resulting from any ideas, methods, instructions or products referred to in the content.
